# DNA Reaction System That Acquires Classical Conditioning

**DOI:** 10.1021/acssynbio.3c00459

**Published:** 2024-01-27

**Authors:** Takashi Nakakuki, Masato Toyonari, Kaori Aso, Keiji Murayama, Hiroyuki Asanuma, Tom F. A. de Greef

**Affiliations:** † Department of Intelligent and Control Systems, Faculty of Computer Science and Systems Engineering, Kyushu Institute of Technology 680-4 Kawazu, Iizuka, Fukuoka 8208502, Japan; ‡ Department of Biomolecular Engineering, Graduate School of Engineering, Nagoya University, Furo-cho, Chikusa-ku, Nagoya 4648603, Japan; § Laboratory of Chemical Biology and Institute for Complex Molecular Systems and Computational Biology Group, Department of Biomedical Engineering, Eindhoven University of Technology, De Zaale, Eindhoven 5600 MB, The Netherlands

**Keywords:** classical conditioning, learning, memory, forgetting, DNA strand displacement

## Abstract

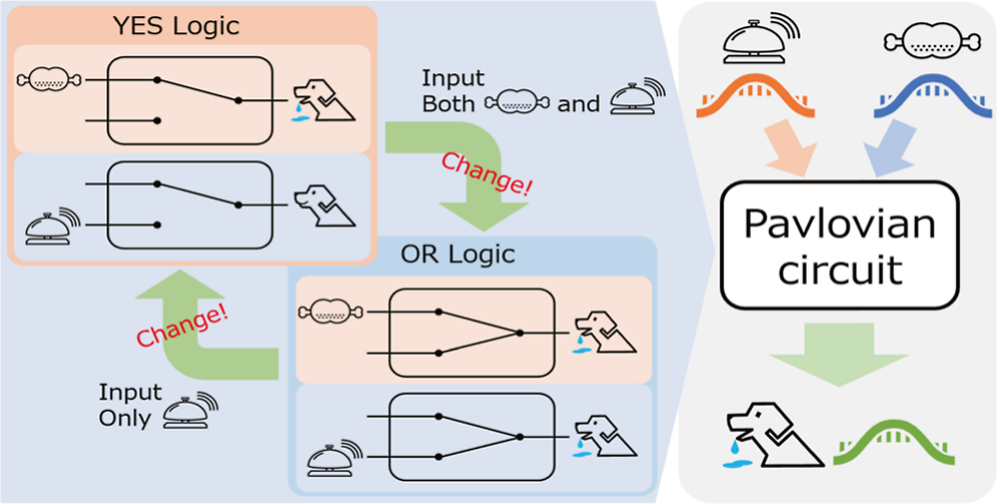

Biochemical reaction networks can exhibit plastic adaptation to alter their functions in response to environmental changes. This capability is derived from the structure and dynamics of the reaction networks and the functionality of the biomolecule. This plastic adaptation in biochemical reaction systems is essentially related to memory and learning capabilities, which have been studied in DNA computing applications for the past decade. However, designing DNA reaction systems with memory and learning capabilities using the dynamic properties of biochemical reactions remains challenging. In this study, we propose a basic DNA reaction system design that acquires classical conditioning, a phenomenon underlying memory and learning, as a typical learning task. Our design is based on a simple mechanism of five DNA strand displacement reactions and two degradative reactions. The proposed DNA circuit can acquire or lose a new function under specific conditions, depending on the input history formed by repetitive stimuli, by exploiting the dynamic properties of biochemical reactions induced by different input timings.

## Introduction

It has recently become possible to construct biochemical reaction systems with intrinsic plastic adaptation capabilities by rationally combining existing basic reaction mechanisms.^1−3^ This capability is based on the versatility of biomolecules, such as proteins,^4^ and the structure and dynamics of biochemical reaction systems.^5^ The principles underlying the plastic adaptability of biochemical reaction systems to their environment represent a crucial issue in academic fields dealing with biochemical reaction systems beyond systems biology.^6^

Recent advances in DNA nanotechnology have enabled methodologies to explore the operating principles of biochemical reaction systems with artificially synthesized nucleic acids to construct specific biomolecular reaction networks.^7^ Systems with plastic adaptation have memory and learning capabilities and have been actively studied in the field of DNA computing over the past decade,^8−11^ as pioneered by Qian et al.^12^ These studies focus primarily on implementing well-established machine learning algorithms, such as neural networks, on DNA reaction systems (hereafter, DNA circuits), while addressing basic learning at the simulation level.^13^ In contrast, an operating principle that can alter the circuit functions depending on the history (stimulus level and timing) of the input stimuli while considering the dynamic properties of DNA circuits has been proposed,^14,15^ implying further possibilities to extend the capability of biochemical reaction systems.^16−20^ However, adaption to DNA circuits with memory and learning capabilities by exploiting the dynamic properties of biochemical reactions remains challenging.^21^

Based on the importance of understanding the operating principles of biochemical reaction systems with intrinsic plastic adaptation capabilities, we aimed to develop a DNA circuit with memory and learning capabilities. In this study, a DNA circuit that acquires classical conditioning as a typical learning task was considered using a simple reaction mechanism in an experimentally feasible manner. Classical conditioning, also known as Pavlovian conditioning,^22^ is a physiological phenomenon based on memory and learning in which the paired presentation of stimuli results in associations between the elements and changes in response. By extending the design concept and fully utilizing the dynamic properties of biochemical reaction systems induced by different input timings,^15^ we constructed a basic DNA circuit that can plastically acquire or forget new functions under a specific condition, depending on the input history formed by repetitive stimuli.

## Results and Discussion

### Circuit Design

The Pavlovian-conditioned reflex is a typical example of classical conditioning.^22^ When food is presented to a dog, the dog unconsciously secretes saliva as a result of a physiological phenomenon known as “unconditioned reflex.” In contrast, ringing a bell without presenting food to the dog does not trigger the unconditioned reflex. However, by repeating trials of ringing the bell while simultaneously presenting food to the dog, the dog begins to salivate upon mere ringing of the bell. This responsive change is known as “conditioned reflex”, a learning mechanism in the brain where food and bells are considered unconditioned and conditioned stimuli, respectively. A recent study elucidated the mechanism of Pavlovian conditioning through a series of murine experiments. In the striatal medium spiny neurons, the transmission efficiency of synaptic (excitatory) signaling with glutamate can plasticly increase at a specific condition, wherein the condition is that reward signaling with dopamine acts on the synaptic signaling within a narrow time window after glutamate activation.^23^ Forgetting the acquired conditioned reflex is also an important mechanism for learning systems. After a conditioned reflex has been established, if only the conditioned stimulus is administered repetitively without the unconditioned stimulus, then the conditioned reflex no longer occurs.

**Figure 1:**
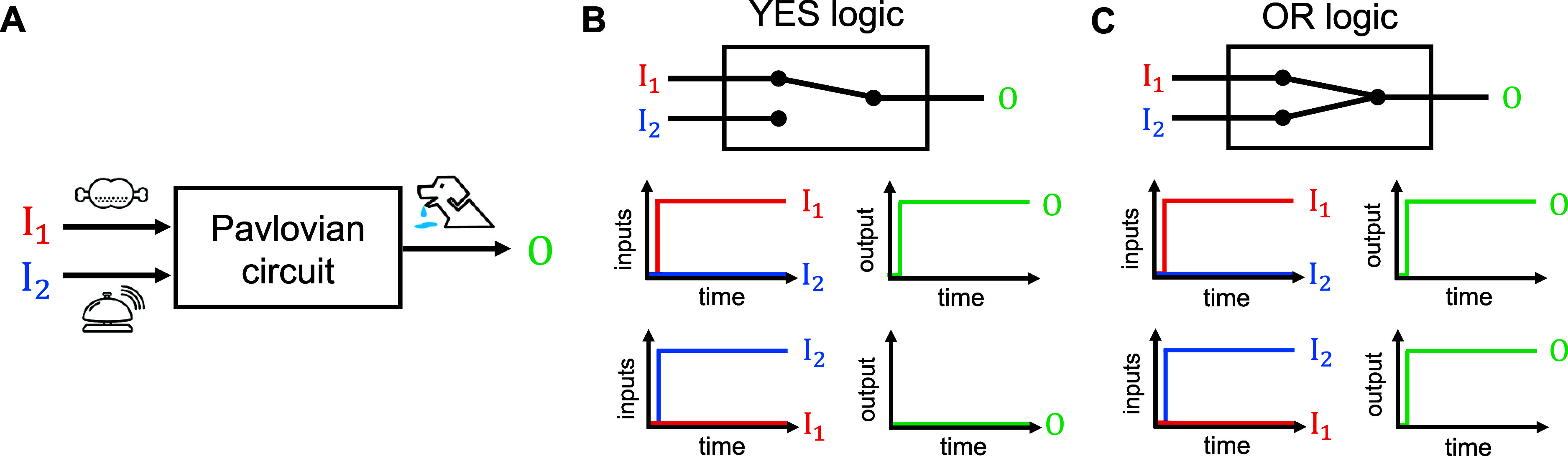
Conditioned reflex circuit. (A) Conditioned reflex circuit is a logical circuit with two inputs, I_1_ and I_2_, and an output, O. (B) In the prelearning condition, the circuit functions as a “YES” gate, where the output O responds to only I_1_ but not to I_2_. (C) In the postlearning condition, the circuit functions as an “OR” gate, where the output O responds not only to I_1_ but also to I_2_.

The learning mechanism in Pavlovian conditioning can be simplified from the perspective of logical operations as follows: consider a two-input and one-output circuit, as shown in Figure 1A, where I_1_ and I_2_ conceptually correspond to “feed-related” and “bell-related” inputs and O “saliva” output, respectively. In the prelearning unconditional reflection, the circuit function corresponds to “YES” logic that outputs only in response to input I_1_ but not to I_2_ (Figure 1B). However, via the learning process in which repeated simultaneous inputs of both I_1_ and I_2_ are provided, in the postlearning conditional reflection, the circuit function alters to “OR” logic that responds not only to I_1_ but also to I_2_ (Figure 1C). Nonetheless, if only input I_2_ is repeatedly applied in the postlearning condition, the output responses to I_2_ gradually weaken; that is, the OR function is gradually lost toward the YES circuit. In this study, we define this plastic, changeable circuit as a “conditioned reflex circuit.”

**Figure 2:**
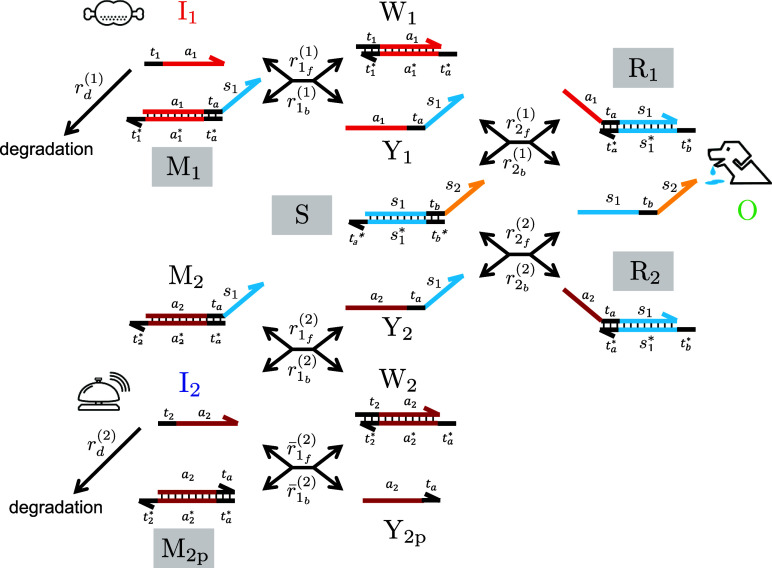
Schematic view of the conditioned reflex circuit using the DNA strand displacement mechanism. The reactions are described with six single-stranded DNAs (I_1_, I_2_, Y_1_, Y_2_, Y_2p_, and O) and eight double-stranded DNAs (M_1_, M_2_, M_2p_, W_1_, W_2_, S, R_1_, and R_2_). Their structures are illustrated with directional arrows; the arrowhead denotes the 3′ end, and the opposite side is the 5′ end. Each DNA strand comprises some domains, where the labels *t*_1_, *t*_2_, *t*_a_, and *t*_b_ are the toehold domains that provide the starting point of the binding in the DNA strand displacement reaction, and the labels *a*_1_, *a*_2_, *s*_1_, and *s*_2_ are the recognition domains that control the linkage of the binding reaction. A double-stranded DNA is illustrated by two opposing arrows with hatching, and domains that have complementary base sequences are indicated by an asterisk (e.g., *t*_1_* and *s*_1_*). The bidirectional arrows connecting the DNA strands denote reversible DNA strand displacement reactions (, , , , , , , , , and ), where the forward and backward reactions are denoted by subscript “*f*” and “*b*”, respectively, and the directional arrows from I_1_ and I_2_ denote the degradation reactions (*r*_*d*_^(1)^ and *r*_*d*_^(2)^). The double-stranded DNAs that have initial concentrations at the prelearning condition are denoted by shadowed, square boxes.

In accordance with the abstracted operation of classical conditioning, we designed the conditioned reflex circuit based on a toehold-mediated DNA strand displacement mechanism (Figure 2), where the chart drawn by Visual DSD^24^ is also provided in Figure S1. The operation principle is summarized as follows:

#### Operation in the Prelearning Condition (Unconditional Reflection)

In the case that only input strand I_1_ is provided (Figure S2A), the first-round strand displacement reaction  and  triggered by the binding of I_1_ with memory gate M_1_ generates an excitation strand Y_1_ and waiting strand W_1_. Subsequently, the second-round strand displacement reaction  and , triggered by the binding of Y_1_ with reservoir gate S, generates an output strand O and reward strand R_1_. Notably, output O also induces a different strand displacement reaction  and  with reward strand R_2_ simultaneously while generating excitation strand Y_2_. In contrast, in the case that only the input strand I_2_ is provided (Figure S2B), the first-round strand displacement reaction  and  does not occur as long as M_2_ has no initial concentration. Instead, another first-round strand displacement reaction  and  between I_2_ and pseudomemory gate M_2*p*_ occurs while generating the pseudoexcitation strand Y_2*p*_ and waiting strand W_2_.

#### Operation during the Learning Process

In a case where I_1_ and I_2_ are provided simultaneously (Figure S2C), all strand displacement reactions occur as explained above. Subsequently, it follows a special condition for learning that *Y*_2_ and *W*_2_ appear simultaneously, and consequently, the strand displacement reaction  triggered by the binding of Y_2_ with W_2_ updates the memory gate concentration M_2_.

#### Operation in the Postlearning Condition (Conditional Reflection)

In the postlearning condition, we assume that a sufficient initial concentration is stored in M_2_. On top of the output response by input I_1_ as with the prelearning condition, even in the case that only I_2_ is applied, the first- and second-round strand displacement reactions (, , , and ) occur with a sufficient amount of M_2_, while generating output strand O and reward strand R_2_.

#### Renewable Mechanism for Responding to Repetitive Inputs

For the learning circuit, a renewable mechanism is essential to respond to repetitive inputs. More precisely, all single- and double-stranded DNAs, except for the memory gate M_2_, must return to their initial concentrations after the output response to the previous input in preparation for the next input. As all strand displacement reactions are designed to be reversible, the circuit can be “renewable” by introducing any adequate reaction mechanism (*r*_d_^(1)^ and *r*_d_^(2)^) to eliminate I_1_ and I_2_, such as a degradation reaction by exonucleases.

### Acquiring Conditioned Reflex

**Figure 3:**
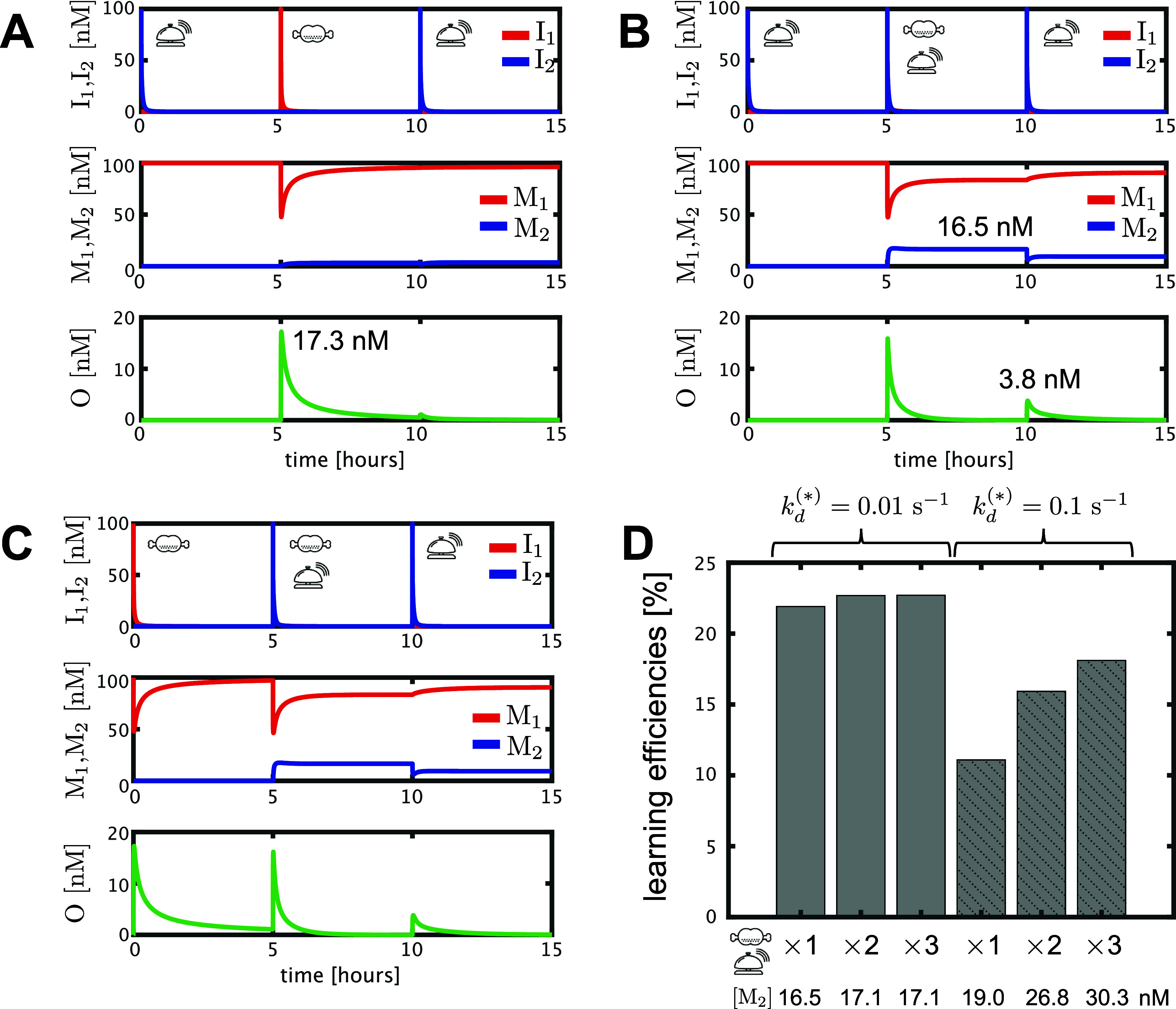
Acquiring conditioned reflexes depending on input patterns. (A–C) The upper, middle, and lower panels show the time-course data of inputs, memory gates, and outputs, respectively. The input patterns are “B–F–B” for (A), “B–FB–B” for (B), and “F–FB–B” for (C). The peak values of interest are denoted in the lower panels of (A,B). (D) Learning efficiencies are calculated by the peak of the I_2_-induced output in the postlearning condition divided by the I_1_-induced output in the prelearning condition, where the I_2_-induced outputs after one, two, and three repetitive, simultaneous inputs were evaluated in two different parameter settings. The accumulated concentrations of M_2_ for input patterns “FB”, “FB–FB”, and “FB–FB–FB” are denoted below the plot. The detailed data are also shown in Figures S6 and S7.

We have verified our design of the conditioned reflex circuit based on numerical simulations, where the mathematical model described by ordinary differential equations, the strand displacement reaction rates , , , and  (*i*, *j* = 1, 2), the degradation rates *k*_d_^(1)^ and *k*_d_^(2)^, and initial concentrations are provided in the Methods section. All of the simulations were performed with Matlab (MathWorks, Inc.). First, we evaluated the operation in the prelearning condition (Figure S2D–F). The output responses appeared in the input conditions of ([I_1_](0), [I_2_](0)) = (100, 0) for (D), ([I_1_](0), [I_2_](0)) = (100, 100) for (F), but not ([I_1_](0), [I_2_](0)) = (0, 100) for (E), where [I*](0) denotes the initial concentration (nM) of strand I* at *t* = 0. In addition, the renewable mechanism by degradation reactions *r*_d_^(1)^ and *r*_d_^(2)^ successfully works to restore the concentration distribution in the reaction system after responses to its initial concentrations, except for the desired accumulation of the memory gate M_2_ in the learning condition ([I_1_](0), [I_2_](0)) = (100, 100) (also see Figures S3–S5 for the time-course data of all strands). Next, we evaluated the operations when various input patterns were applied to the circuit to confirm that its function is plastically altered only under the learning condition. For the sake of simplicity of notation, we denoted the input pattern, which comprised multiple consecutive inputs in the timeline, by a character string formed by concatenating “F” as “feed-related” I_1_ and “B” as “bell-related” I_2_. For example, input pattern F–B–F represents three consecutive inputs of I_1_, I_2_, and I_1_ over time. In addition, simultaneous inputs are denoted by “FB.” In the case of the input pattern “B–F–B”, which does not satisfy the learning condition, the first input I_1_ induced a strong output (17.3 nM of peak), but the following input I_2_ did not trigger the responses as the concentration of memory gate M_2_ was not increased noticeably (Figure 3A). However, in the case of patterns “B–FB–B”, which satisfy the learning condition, although the first-time I_2_ did not trigger the response, the second-time I_2_ successfully induced the output response (3.8 nM of peak) as the simultaneous inputs I_1_ and I_2_ led to an increase in M_2_ concentration (16.5 nM) during the period of resetting (Figure 3B). Other learning conditions, such as “F–FB–B” also induced a reasonable “update” of memory gate M_2_ (Figure 3C). In terms of learning efficiency, the peak of the I_2_-induced output in the postlearning condition was approximately 22.0% (=3.8/17.3 × 100) of the I_1_-induced output in the prelearning condition, as indicated in Figure 3A,B. In particular, the learning efficiencies were not markedly increased even if several simultaneous inputs were applied to the circuit. This can be explained in terms of the update width of the memory gate M_2_, where the accumulated concentrations of M_2_ after repetitive “FB” inputs were 16.5, 17.1, and 17.1 nM for input patterns “FB”, “FB–FB”, and “FB–FB–FB”, respectively (Figure 3D). The update width of M_2_ is dependent on the gate concentrations and degradation rate. For example, by doubling the gate concentrations ([M_1_](0) = [M_2p_](0) = [S](0) = [R_2_](0) = 200 nM) and increasing the degradation rate *k*_d_^(^*^)^ by a factor of 10, M_2_ can be updated from 19.0 to 30.3 nM in a stepwise manner (Figure 3D). It should be noted that the output property of the conditioned reflex circuit can be suitably reprocessed by various methods, depending on the applications. For example, as discussed below, the output amplitude can be considerably enhanced by adding a threshold gate to the circuit.

### Forgetting Conditioned Reflex

**Figure 4:**
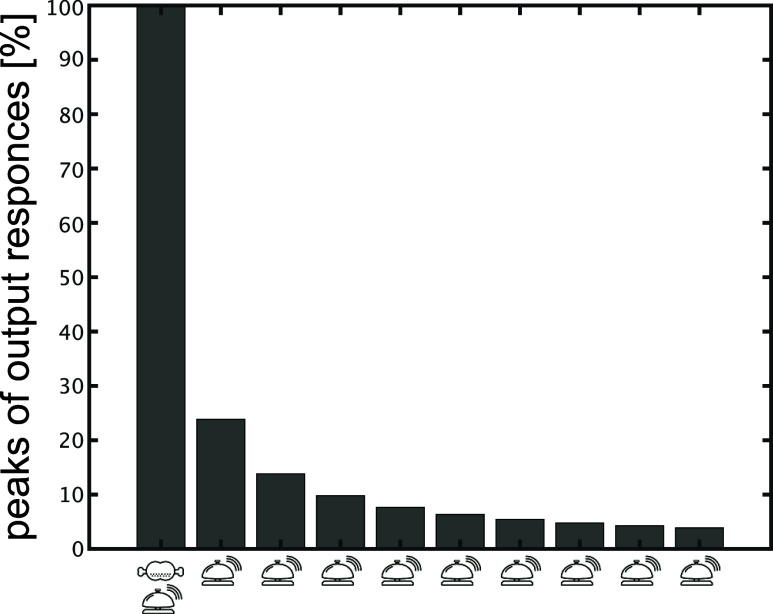
Forgetting the conditioned reflex. The peak of the output response for each input of the input pattern “FB–B–B–B–B–B–B–B–B–B” is calculated, where the peaks are normalized by that of the first “FB” input case. Detailed data are shown in Figure S9.

The conditioned reflex was first acquired by the simultaneous input of I_1_ and I_2_; then, only the I_2_ input was applied nine times repetitively (that is, we applied the input pattern “FB–B–B–B–B–B–B–B–B–B”) (Figure 4). The accumulated concentration of M_2_ and the output response peaks gradually decreased (Figure S8) as the repetitive I_2_ inputs were provided. The speed of forgetting is dependent on gate concentration and degradation rate. In fact, a more rapid decrease in peaks of output responses was observed in the circuit with the setting [M_1_](0) = [M_2*p*_](0) = [S](0) = [R_2_](0) = 200 nM, [R_1_](0) = 50 nM, and *k*_d_^(1)^ = *k*_d_^(2)^ = 0.1 s^–1^ (Figure S10), where the initial dumping of output peak with I_2_ input was approximately twice as large.

### Generalization of the Conditioned Reflex Circuit

**Figure 5:**
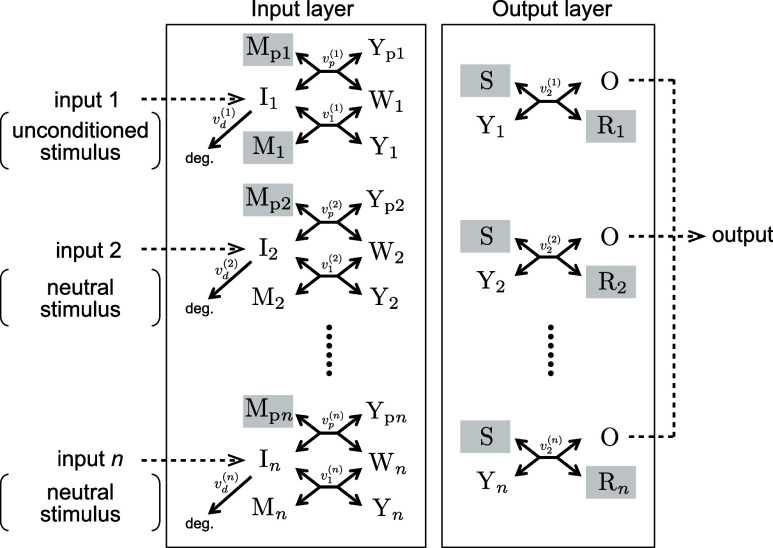
Generalization of the conditioned reflex circuit. Input I_1_ is designed as an unconditioned stimulus that induces the output response, and others I_2_,···,I_*n*_ are designed as neutral stimuli that do not induce the output response in the prelearning condition. The double-stranded DNAs that have initial concentrations at the prelearning condition are denoted by shadowed, square boxes.

In general, classical conditioning is a learning task defined for multiple-input systems. In this section, we consider extending the two-input conditioned reflex circuit to a multiple-input counterpart. Figure 5 shows a schematic view of a generalized version of the conditioned reflex circuit with *n*-input channels. For simplicity of notation, let a combination of *n* inputs (I_1_, I_2_,···, I_*n*_) simultaneously applied to the circuit at a certain time be defined by , where *i*_*k*_ is 1 if the input I_*k*_ is included in the combination and otherwise 0. For example, if inputs I_1_, I_3_, and I_*n*_ are simultaneously applied to the circuit, we denote the combination of inputs as . The learning task for the multiple-input version of classical conditioning is defined as Consider the n-input and 1-output circuit (Figure 5), where the input I_1_ and others I_2_,···,I_*n*_ are designated as the unconditioned stimulus being responsive and neutral stimuli being nonresponsive at the initial state in the prelearning condition, respectively. Assume that two consecutive inputs  and  are applied to the circuit at an appropriate time interval; for the first input , a combination of I_1_,I_*c*1_,I_*c*2_,···,I_cm_ is employed, where *c*_1_, *c*_2_, ..., *c*_m_ ∈ {2, 3, ..., *n*} (*m* ≤ *n* – 1), and *m* is a positive integer. Then, the circuit is called a “generalized conditioned reflex circuit” if the output O is responsive only upon  such that at least one of {*i*_1_, *i*_c1_, *i*_c2_,···, *i*_cm_} is 1 and the others are zero. We performed the numerical experiments on the generalized conditioned reflex circuit with four input channels, for which the mathematical model and the parameters are provided in the Methods section. Since we assume simultaneous inputs with the unconditioned stimulus I_1_ for  in prelearning condition, there are eight possible combinations for . According to the output responses for all 32 input histories when only one input was applied for  in the postlearning condition, we can see that the output O was responsive in the case of the 12 input histories that were expected to obtain the conditioned reflex, in addition to the trivial eight input histories shown in the first column (Figure S11). We also confirmed that simulations of all 120 possible input histories, including the 32 cases, agreed with the expected results (Figure S12). Since the generalized conditioned reflex circuit consists of a minimal number of reaction mechanisms, the learning efficiency tends to decrease as the number of input channels increases (See Figure S13 for the 10-input case). However, the dynamic behaviors qualitatively demonstrate that the conditioned reflexes were acquired by this minimal mechanism.

### Discussion 1: Binarization of the Output Response by Thresholding

**Figure 6:**
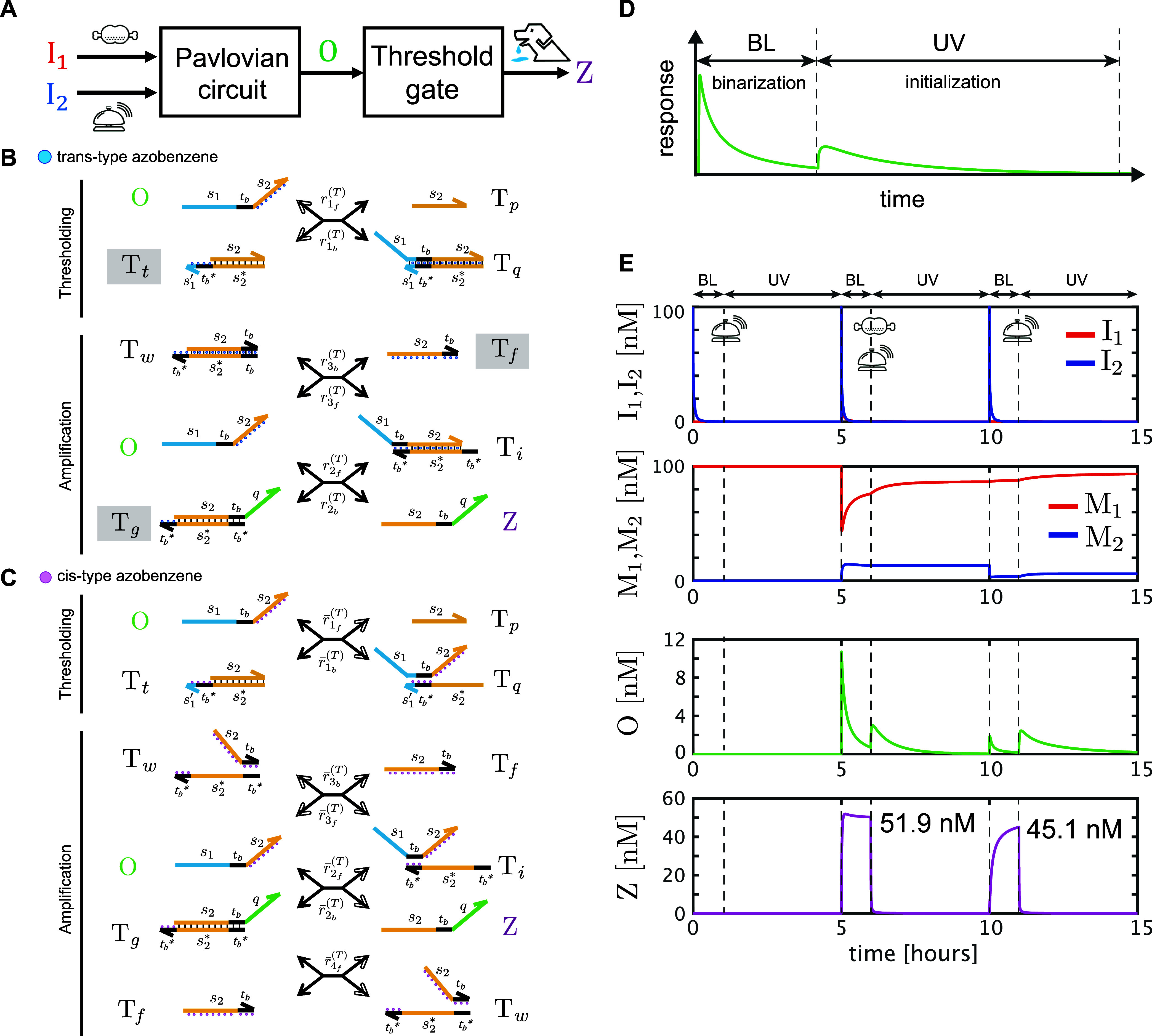
Binarization of output response by thresholding. (A) Threshold gate is connected to the conditioned reflex circuit. (B,C) Schematic views of the threshold gate under blue light (BL) irradiation (B) and ultraviolet (UV) irradiation (C) conditions, where the reactions are comprised of four single-stranded DNAs (O, T_p_, T_f_, and Z) and five double-stranded DNAs (T_t_, T_q_, T_g_, T_i_, and T_w_) and are denoted by the bidirectional arrows connecting the DNA strands in the same notation as in Figure 2, except that considerably slow reactions are represented by white arrows. Small circles attached to domains denote azobenzene modifications, where blue ones in (B) and purple ones in (C) are *trans*- and *cis*-type azobenzene, respectively. Double-stranded DNAs with initial concentrations are denoted by shadowed, square boxes. Corresponding ordinary differential equations, the initial concentrations, and the reaction rates are described in Text S3. (D) Timing chart of the photoresponsive control. BL or UV was irradiated alternately. (E) First, second, third, and fourth panels show the time-course data of inputs, memory gates, net output of the conditioned reflex circuit, and binarized output of the threshold gate, respectively. The peak values of Z upon the “FB” and the second “B” inputs are denoted in the fourth panel.

The conditioned reflex circuit in Figure 2 was designed with the minimal structure required to meet the specifications. Depending on practical applications, appropriate mechanisms can be added to reprocess the output property of the conditioned reflex circuit. Following, we demonstrate a binarization of output response using a threshold gate that can distinguish between logically low and high in the output with reference to a threshold level. Let the threshold gate be connected to the conditioned reflex circuit as shown in Figure 6A, and observe the new output, Z. In this demonstration, we employed a seesaw gate^25^ as a thresholding mechanism comprising thresholding ( and ) and amplification mechanisms (, , , and ) in Figure 6B. Since the conditioned reflex circuit must respond to repetitive inputs for learning, the seesaw gate is also required to be renewable. Here, we adopt a renewable design with the photoresponsive molecule, “azobenzene.” A photoresponsive control design using the azobenzene modification of DNA strands can make DNA circuits renewable.^26,27^ Azobenzene can exist in two structures, the trans and cis forms, which can be interconverted under ultraviolet (UV) and blue light (BL) irradiation, respectively.^28^ When azobenzene is incorporated into the DNA base sequence, the stability of the double-helix structure can be controlled by light irradiation. Specifically, under BL irradiation, the trans form stabilizes the double-stranded structure, while under UV irradiation, the cis form destabilizes it. This property of azobenzene can be applied to DNA strand displacement reactions by modifying the toehold and recognition domains with azobenzene, which can alter the balance between forward and backward reaction flows by using BL/UV irradiation. In this demonstration, we performed photoresponsive control such that the thresholding executed the binarization of output responses from the conditioned reflex circuit under BL or was initialized toward the initial concentration under UV irradiation (Figure 6D). In accordance with the renewable design proposed by Tamba et al.,^27^ domains *s*_2_, *s*_1_′*t*_b_*, *t*_b_*, and *s*_2_*t*_b_ of O, T_t_, T_g_, and T_f_, respectively, were modified with azobenzene, where as much azobenzene as possible was inserted in these domains (Figure 6B,C). Under BL irradiation (in Figure 6B), all reactions , and  occurred based on the usual strand displacement mechanism, meaning that the gate functioned as a threshold gate. For UV irradiation (Figure 6C), a set of reactions , , and  became dominant, and as a consequence, all reactions of the threshold gate proceeded in the direction of the initial concentrations, indicating that the gate was initialized. Figure 6E shows the simulation results in the case of input patterns “B–FB–B”, which satisfy the learning condition; the initial concentrations of the threshold gate were given by [T_t_](0) = [T_g_](0) = [T_f_](0) = 100 nM (others 0), and irradiation durations were set at 1 and 4 h for BL and UV, respectively. The threshold gate enhanced the net output responses from the conditioned reflex circuit upon the “FB” and the second “B” inputs and was effective in logically distinguishing between high and low.

### Discussion 2: Synchronization in Learning Conditions

**Figure 7:**
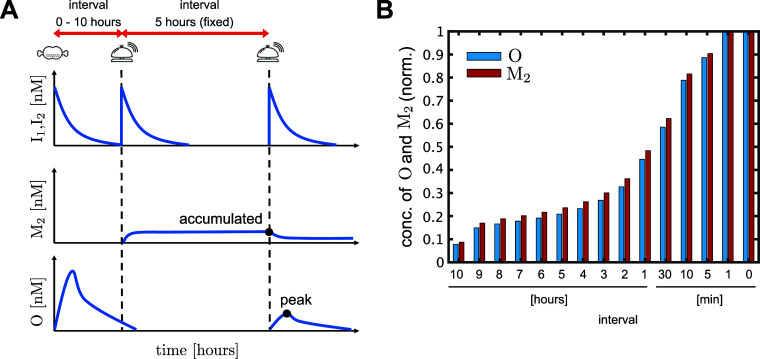
Synchronization in learning conditions (A) Interval between the first and second inputs of the input pattern “F–B–B” were changed from 0 to 10 h, where 0 min represents the simultaneous inputs of I_1_ and I_2_, and the interval between the second and third inputs was fixed at 5 h. The accumulated concentrations of M_2_ after the second input and the peaks of output O after third input were investigated. (B) Simulation results of the accumulated M_2_ concentrations and the peaks of the output O are shown by red- and blue-colored bars, respectively. The vertical axis is normalized by the concentrations in the simultaneous input case.

It has been reported that the learning principle of Pavlovian conditioning in the brain revealed the importance of activation synchronization induced by unconditioned and conditioned stimuli within a narrow time window.^23^ In our design, the condition for a memory gate M_2_ to be updated is the simultaneous existence of the I_1_-induced Y_2_ strand and the I_2_-induced W_2_ strand, as shown in Figure S2C. Hence, as long as the time of the I_1_ and I_2_ inputs is within a time window, the learning condition is expected to be satisfied. As shown in Figure 7, the accumulated M_2_ concentration decreased monotonically as the interval between the I_1_ and I_2_ inputs became longer. Consequently, the peak of the output response also had a similar profile as that of the interval. Therefore, our design reproduces the properties of synchronization in Pavlovian conditioning.

### Discussion 3: Implications for Functional Enhancements

As can be seen, the conditioned reflex circuit consists of five DNA strand displacement reactions and two degradation reactions. Provided that the degradation reactions are also implemented by an enzyme-free mechanism, the entire circuit becomes a completely enzyme-free system. For example, an enzyme-free degradation mechanism based on DNA strand displacement reactions is available^29^ and has been applied to various circuits.^27,30^ As discussed in an earlier study,^7^ enzyme-free designs allow us to analyze/design the reaction systems in detail and perform mathematical modeling based on the well-defined DNA strand displacement mechanism without black-box mechanisms. In addition, it would be beneficial to take the enzyme-free design in some situations such that the conditioned reflex circuit is connected to other enzyme-free circuits or experimental constraints (buffer composition, temperature, pH) are appropriate for an enzyme-free system.

However, if the entire reaction system, including the DNA strand displacement cascades, degradation reactions, and thresholding gates, can be reconstructed based on enzymatic reaction design such as PEN toolbox^31,32^ while assuring renewability, it might be possible to enhance reaction speed and robustness and further simplify the circuit.^7,33^

Regarding the freedom of input channels of the conditioned reflex circuit, the generalized version of the circuit can receive multiple kinds of input strands but still requires prescribing the base sequence for each input strand. To deal with arbitrary input sequences rather than a preset of sequences, one might consider a polymerase-based primer exchange reaction to generate user-specified sequences from primer strands applied to the circuit.^34^

## Conclusions

In this study, we explored the operating principle of biochemical reaction systems with intrinsic plastic adaptation capabilities and designed a DNA circuit that possesses classical conditioning. The designed circuit was simply constructed with a maximum of five DNA strand displacement reactions and two degradation reactions. Taking advantage of the dynamical properties in the DNA circuit based on the input history of the kind and timing, as suggested by the learning principles of the Pavlovian-conditioned reflex, provides freedom in DNA circuit design.

Our design sought to address the design issue in the simplest manner to improve further the performance, including the efficiency, robustness, and reliability of the learning system. However, according to the required specifications for an application, incorporation of various technical ingenuities from the sequence, molecular, and platform levels is needed, such as the introduction of clamp domains,^25^ artificial nucleic acids,^35^ or enzymatic reaction mechanisms, including the PEN DNA toolbox^31^ and the BIO-PC system.^36^

If considering a direct application in future studies, the conditioned reflex circuit designed herein provides a logic circuit in which the YES and OR functions are switched reversibly according to the input condition. For example, by creating a tree-structured network by connecting the two-input and one-output YES/OR switching circuits in a modular manner, it is proposed that more intelligent circuits will be designed that are capable of acquiring various functions by learning.^37^

## Methods

The mathematical model of the conditioned reflex circuit shown in Figure 2 is given by ordinary differential equations based on chemical kinetics (Text S1). All reaction rates and the initial concentrations of the conditioned reflex circuit were estimated as follows.

Let the peak and steady-state values of the output response induced by the simultaneous I_1_ and I_2_ inputs be represented by *J*_pk_^(1,2)^ and *J*_ss_^(1,2)^. Similarly, the peak and steady-state values of the output response induced by the I_2_ input are represented by *J*_pk_^(2)^ and *J*_ss_^(2)^. Following the specifications of the conditioned reflex circuit defined in the “Circuit design” section, we consider the output responses induced by the simultaneous I_1_ and I_2_ inputs at the prelearning condition and the I_2_ input at the postlearning condition, as shown in Figure S14. The cost function employed for the parameter estimation of the conditioned reflex circuit is designed by
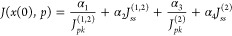
1where *x*(0) is the initial concentration vector and  is the parameter vector comprising reaction rates (also see Text S1). The nonlinear optimization problem of parameter estimation is then formulated as follows: Minimize the cost function *J* subject to the initial concentration *x*(0) and the kinetic parameter *p*. Generally, the reaction rates depend on the toehold lengths involved in a strand displacement reaction and the reaction temperature and, therefore, have distinct values depending on these conditions. Hence, when evaluating the cost function, the lengths of toeholds *t*_1_, *t*_2_, *t*_*a*_, and *t*_*b*_ were searched in the range of 3–6, respectively, and *p* was calculated according to the calculation method,^38^ where 100 nM inputs are applied to the circuit, lengths of the recognition domains *a*_1_, *a*_2_, *s*_1_, and *s*_2_ are fixed by 20 nt, and temperature is assumed to be 37 °C. We successfully determined that the optimal initial concentrations were [M_1_](0) = [M_2p_](0) = [S](0) = [R_2_](0) = 100 nM, [R_1_](0) = 50 nM, and the others were 0 nM; the degradation rates were *k*_d_^(1)^ = *k*_d_^(2)^ = 0.01 1/s, and the optimal lengths of toeholds *t*_1_, *t*_2_, *t*_*a*_, and *t*_*b*_ were 5 nt, where the “ga” (genetic algorithm) library of Global Optimization Toolbox in Matlab was used, and the estimated values were rounded to one significant digit. Then, all strand displacement reaction rates, , , , and  (*i*, *j* = 1, 2) were calculated as 5.32 × 10^–4^ 1/nMs with 164 nM in the critical concentration. The validity of the estimated parameter values was further evaluated by using the sensitivity analysis in Text S4. The mathematical model of the generalized conditioned reflex circuit shown in Figure 5 is also given in Text S2.

## Supporting Information Available

Figures regarding the conditioned reflex circuit drawn by Visual DSD, operating and learning principles, simulations of the acquisition and forgetting of conditioned reflexes under different conditions, simulations of the generalized conditioned reflex circuit, the cost function of the parameter estimation, mathematical modeling of the (generalized) conditioned reflex circuit and the renewable threshold gate, and description of optimization of learning efficiency (PDF)
