# Supplementary material for: DNA Reaction System That Acquires Classical Conditioning
Source: ACS Synth Biol. 2024 Jan 27;13(2):521–9. doi: 10.1021/acssynbio.3c00459 (PMC10877613; doi:10.1021/acssynbio.3c00459)
Supplement: Supplementary file 1 — sb3c00459_si_001.pdf [file sb3c00459_si_001.pdf]

Supporting Information: DNA reaction system that acquires classical conditioning

Takashi Nakakuki,*,†,§ Masato Toyonari,† Kaori Aso,† Keiji Murayama,‡ Hiroyuki Asanuma,‡ and Tom F. A. de Greef¶

†*Department of Intelligent and Control Systems, Faculty of Computer Science and Systems Engineering, Kyushu Institute of Technology*

‡*Department of Biomolecular Engineering, Graduate School of Engineering, Nagoya University*

¶*Laboratory of Chemical Biology and Institute for Complex Molecular Systems and Computational Biology Group, Department of Biomedical Engineering, Eindhoven University of Technology*

§*680-4 Kawazu, Iizuka, Fukuoka 8208502 Japan*

E-mail: nakakuki@ics.kyutech.ac.jp

Phone: +81 (0)948 297716

Supporting Information Available

- Supporting Figure S1: The conditioned reflex circuit drawn by Visual DSD
- Supporting Figure S2: Operating and learning principles
- Supporting Figure S3: Simulation results in case of $([\mathrm{I}_1](0), [\mathrm{I}_2](0)) = (100, 0)$
- Supporting Figure S4: Simulation results in case of $([\mathrm{I}_1](0), [\mathrm{I}_2](0)) = (0, 100)$.
- Supporting Figure S5: Simulation results in case of $([\mathrm{I}_1](0), [\mathrm{I}_2](0)) = (100, 100)$
- Supporting Figure S6: Simulation results with input patterns of "food (F) bell (B)-B" (A), "FB-FB-B" (B), and "FB-FB-FB-B" (C)
- Supporting Figure S7: Simulation results regarding the learning efficiencies
- Supporting Figure S8: Simulation results regarding forgetting the conditioned reflex
- Supporting Figure S9: Simulation results regarding forgetting the acquired function
- Supporting Figure S10: Simulation results regarding forgetting the acquired function with another setting
- Supporting Figure S11: Simulations of the generalized conditional reflection circuit with 4-input channels
- Supporting Figure S12: Simulation results of 4-input and 1-output conditioned reflex circuit
- Supporting Figure S13: Simulation results of 10-input and 1-output conditioned reflex circuit
- Supporting Figure S14: Definition of evaluation values ($J_{pk}^{(1,2)}$, $J_{ss}^{(1,2)}$, $J_{pk}^{(2)}$, and $J_{ss}^{(2)}$) in output responses for designing the cost function of the parameter estimation

- Supporting Figure S15: Efficiency in learning conditions
- Supporting Text S1: Mathematical modeling of conditioned reflex circuit
- Supporting Text S2: Mathematical modeling of generalized conditioned reflex circuit
- Supporting Text S3: Mathematical modeling of the renewable threshold gate
- Supporting Text S4: Optimization of learning efficiency

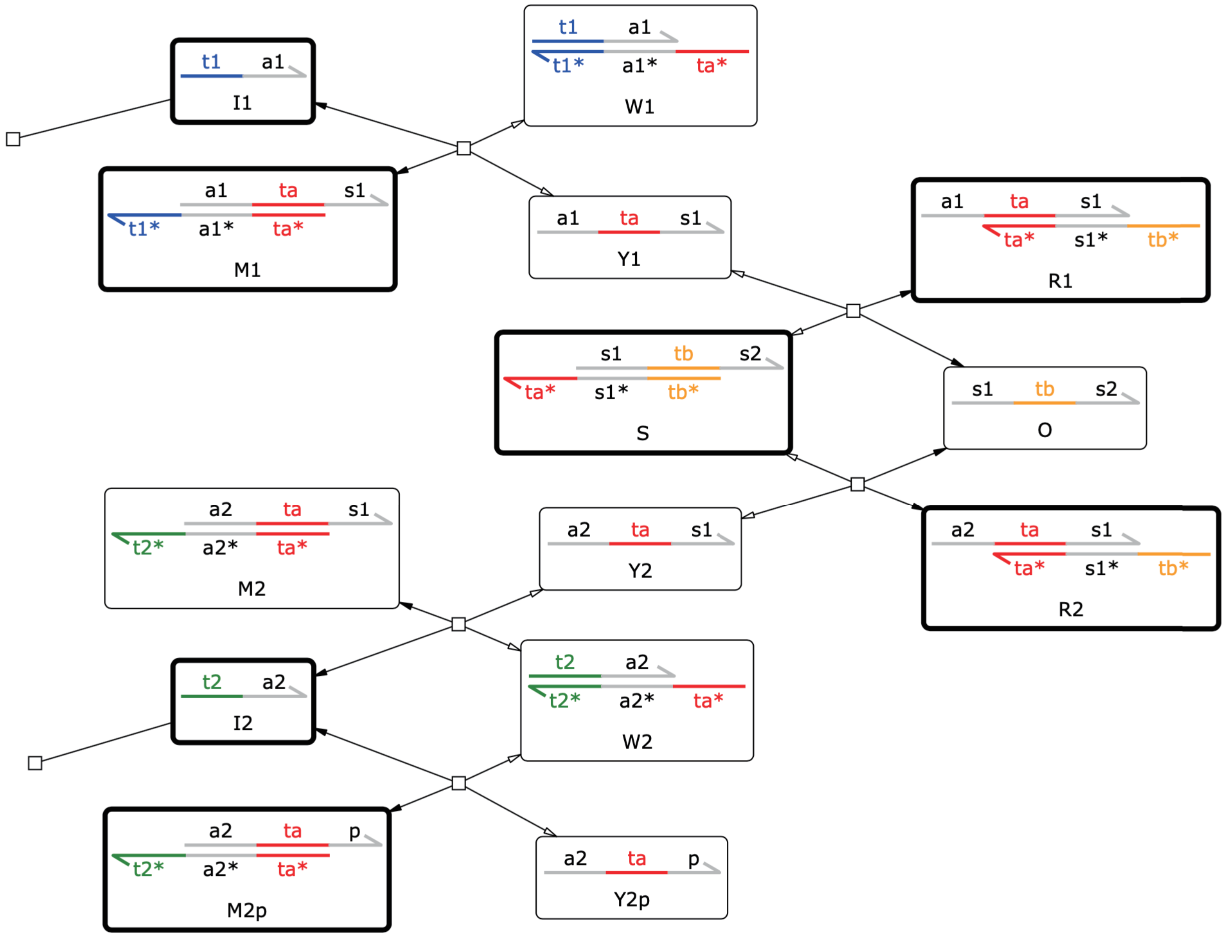

Supporting Figure S1 The chart drawn by Visual DSD.[1] The code is available on GitHub at https://github.com/SYSBIOKYUTECH/conditioned-reflex-circuit.

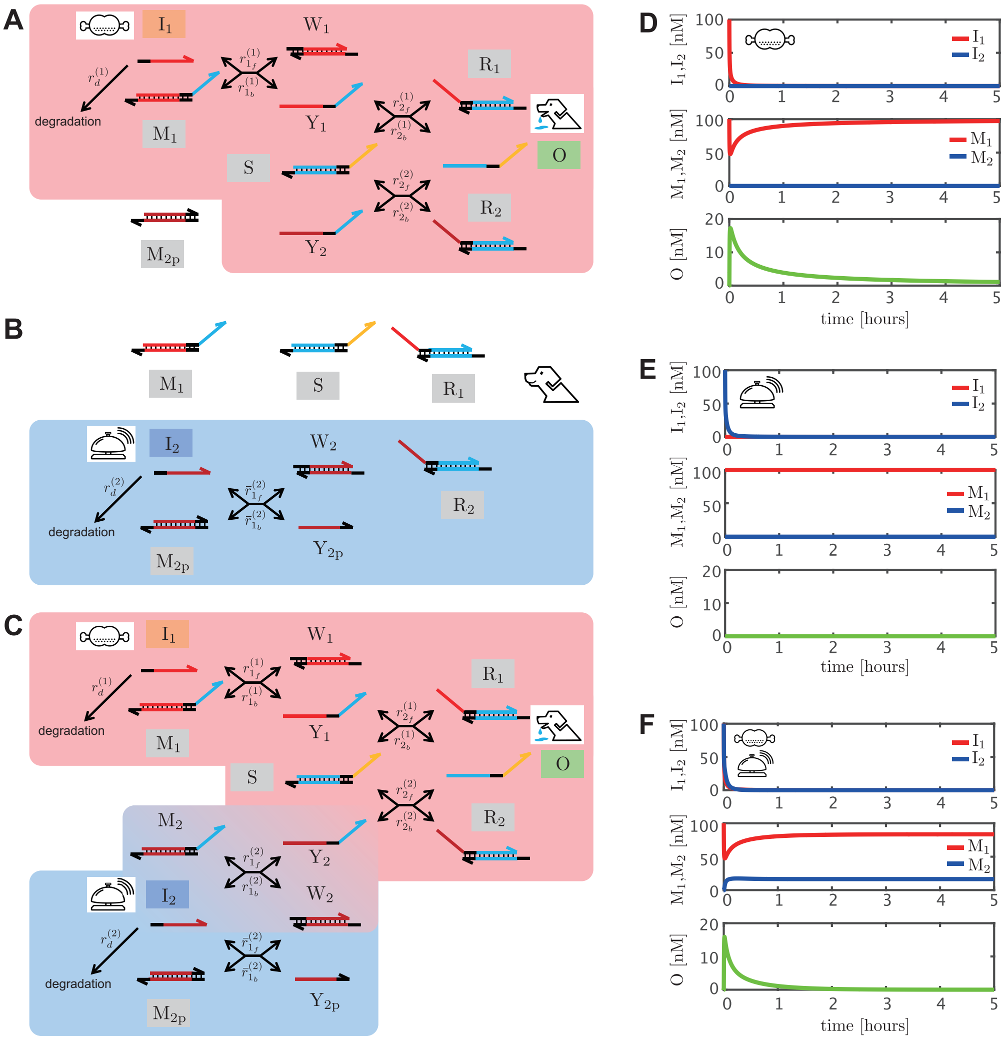

Supporting Figure S2 Operating and learning principles. (A) Reactions that occur on input I_1 are highlighted by a red box. (B) Reactions that occur on input I_2 are highlighted by a blue box. (C) Reactions that occur on simultaneous inputs I_1 and I_2 are highlighted by a colored box, where reactions $r_{1_f}^{(2)}$ and $r_{1_b}^{(2)}$ are represented by a gradient color as they are induced by the fusion of red- and blue-colored reactions. (D-F) Simulation results correspond to the conditions (A–C) in the pre-learning condition, where the upper, middle, and lower panels show the time-course data of inputs, memory gates, and outputs, respectively. Input conditions (nM) are $([I_1](0), [I_2](0)) = (100, 0)$ for (D), $([I_1](0), [I_2](0)) = (0, 100)$ for (E), and $([I_1](0), [I_2](0)) = (100, 100)$ for (F).

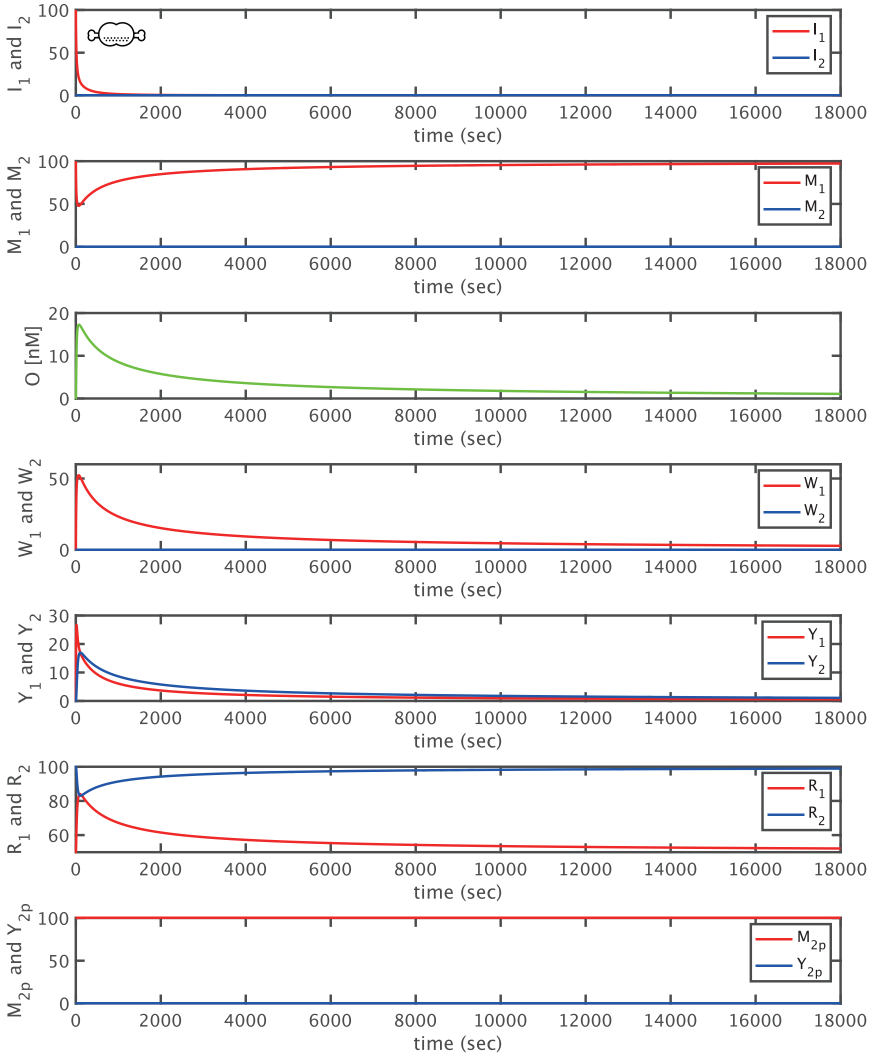

Supporting Figure S3 Simulation results in case of $([\mathrm{I}_1](0), [\mathrm{I}_2](0)) = (100, 0)$.

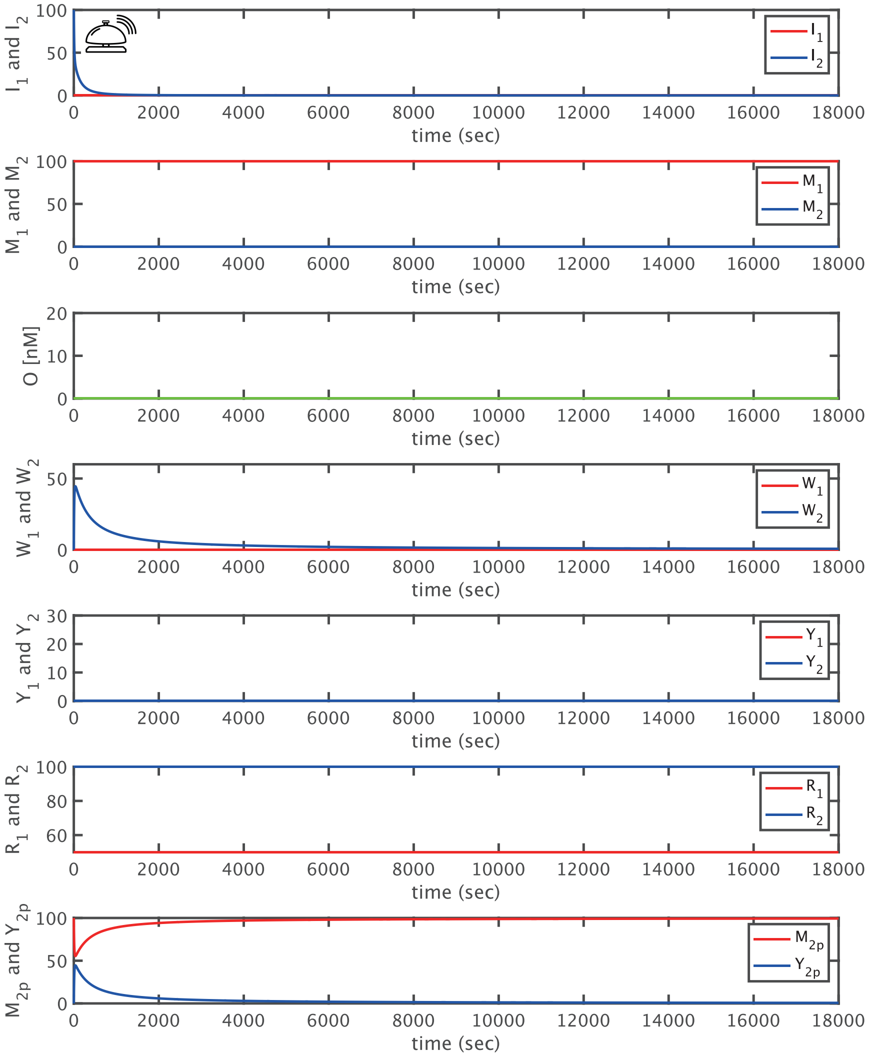

Supporting Figure S4 Simulation results in case of $([\mathrm{I}_1](0), [\mathrm{I}_2](0)) = (0, 100)$.

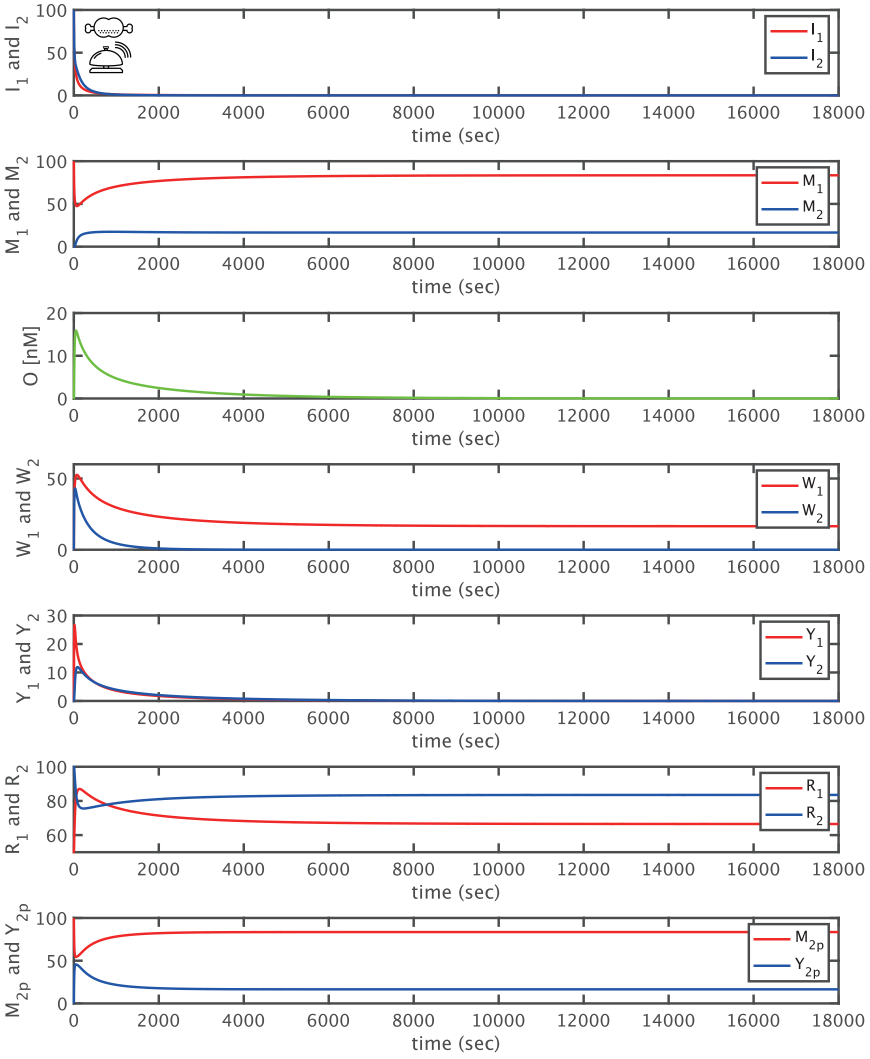

Supporting Figure S5 Simulation results in case of $([\mathrm{I}_1](0), [\mathrm{I}_2](0)) = (100, 100)$.

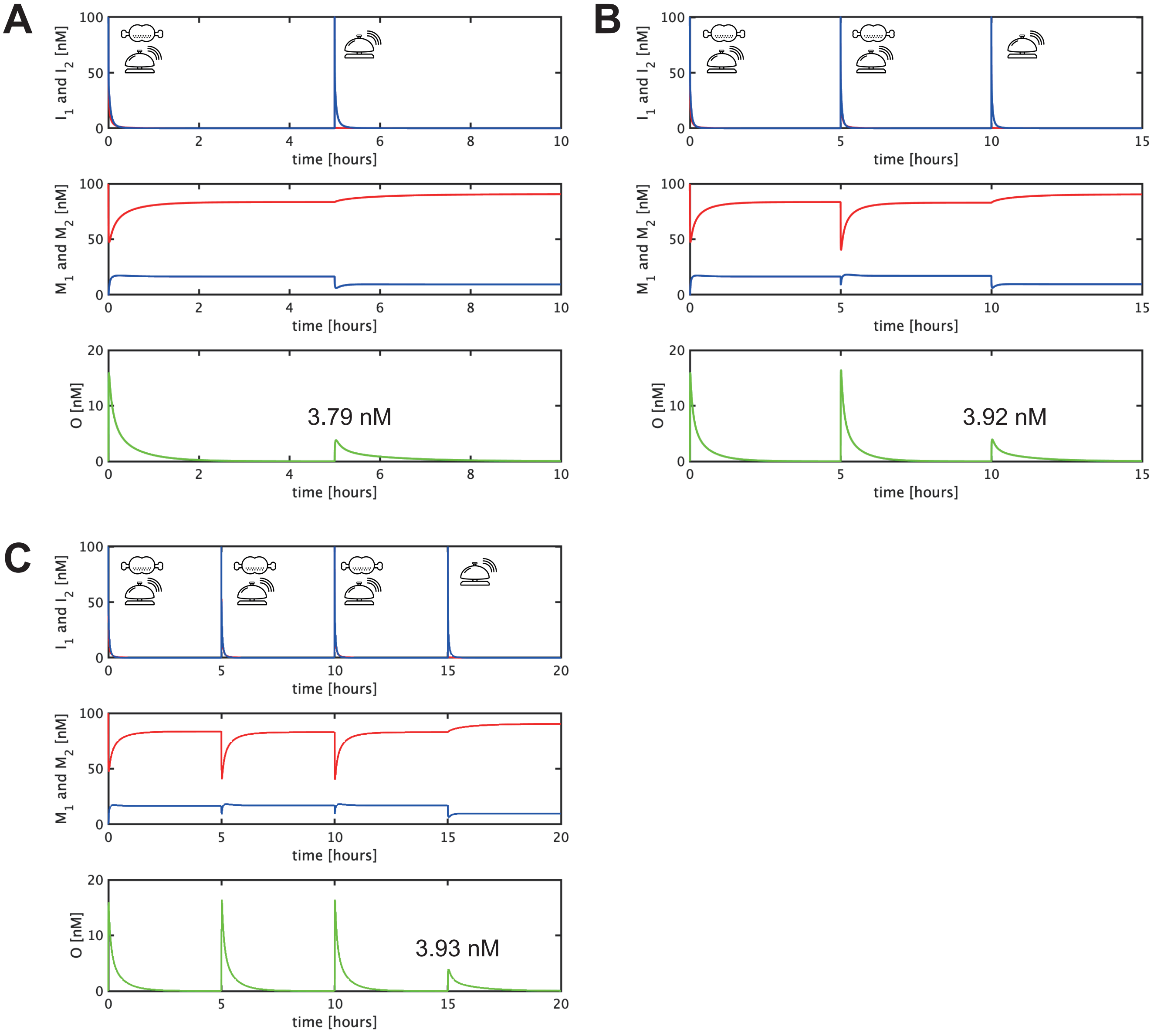

Supporting Figure S6 Simulation results with input patterns of "food (F) bell (B)-B" (A), "FB-FB-B" (B), and "FB-FB-FB-B" (C)

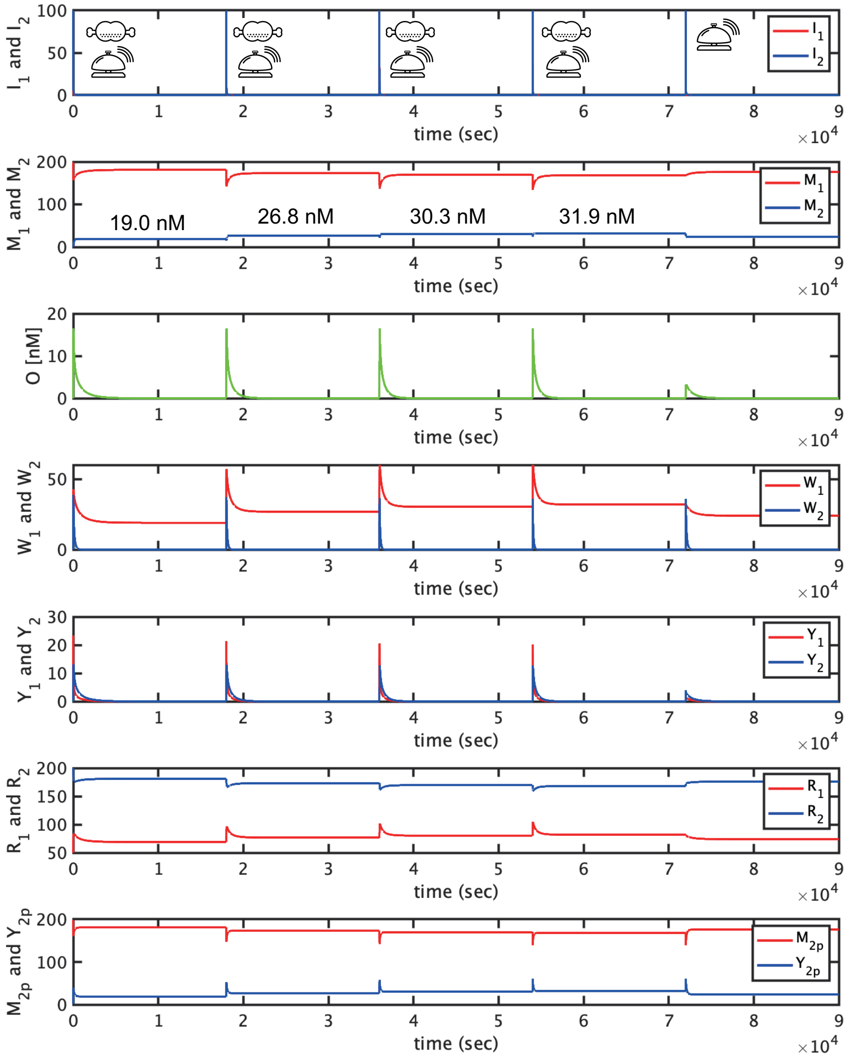

Supporting Figure S7 Simulation results regarding the learning efficiencies. Four repetitive and simultaneous inputs were applied to the circuit with $[M_1](0)=[M_{2p}](0)=[S](0)=[R_2](0)=200$ nM, $[R_1](0)=50$ nM, and $k_d^{(1)} = k_d^{(2)} = 0.1$ s^{-1}. The M_2 concentrations updated for each input are indicated in the plot.

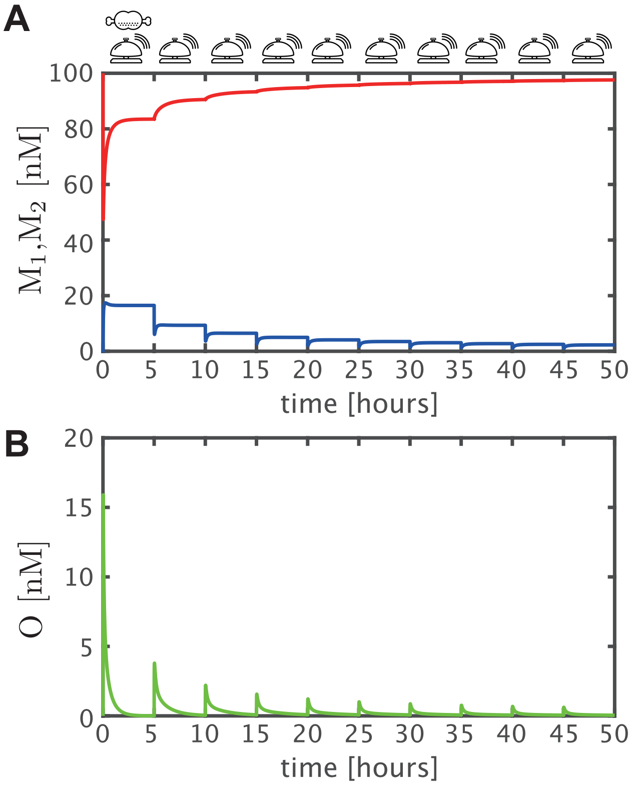

Supporting Figure S8 Simulation results regarding forgetting the conditioned reflex. The time-course data of memory gates (A) and output (B) with input patterns are "FB-B-B-B-B-B-B-B-B-B." The detailed data are also shown in Fig. S9.

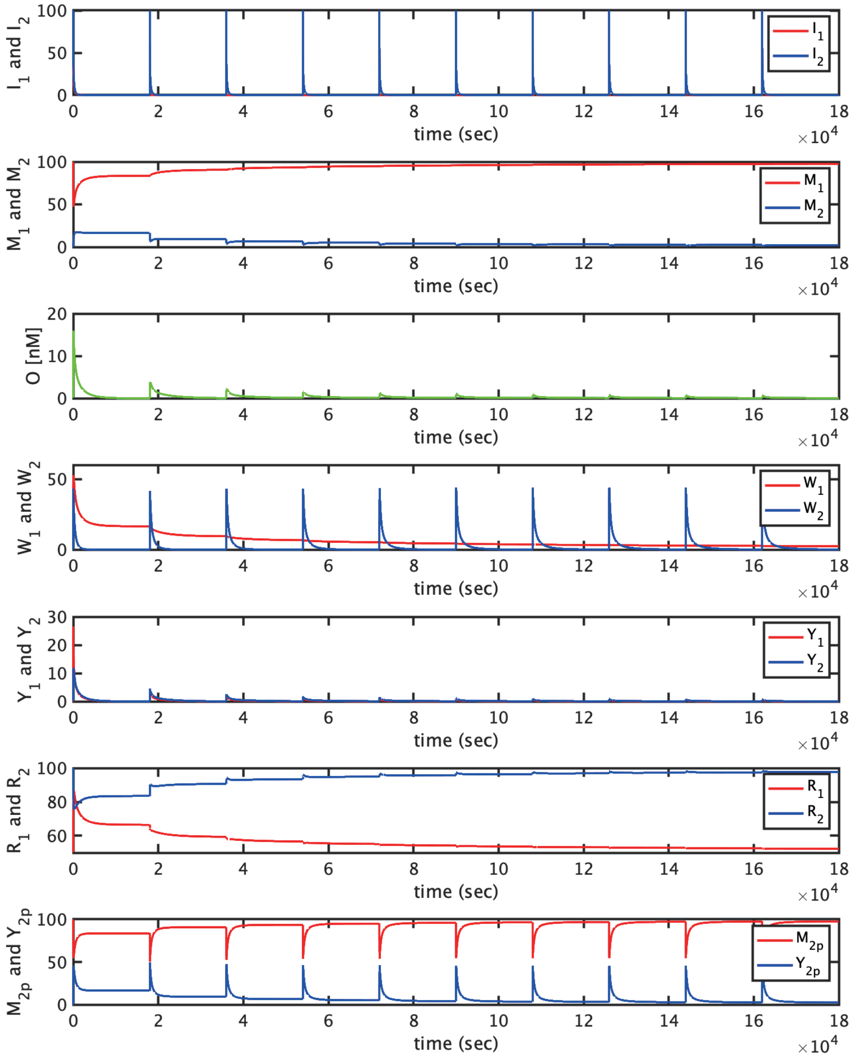

Supporting Figure S9 Simulation results regarding forgetting the acquired function. Ten repetitive input patterns "food (F) bell (B)-B-B-B-B-B-B-B-B-B" were applied to the circuit.

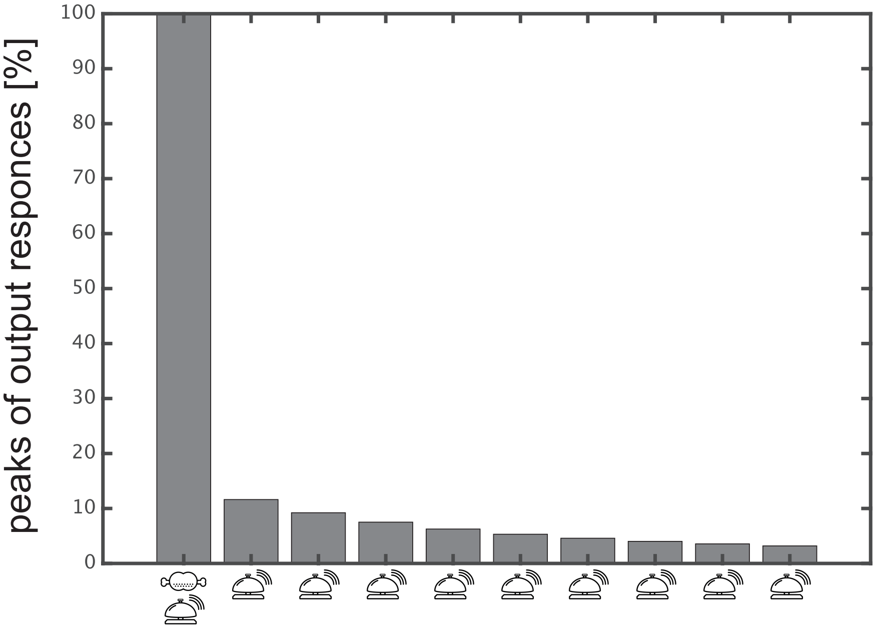

Supporting Figure S10 Simulation results regarding forgetting the acquired function with the setting $[\mathrm{M}_1](0)$=$[\mathrm{M}_{2p}](0)$=$[\mathrm{S}](0)$=$[\mathrm{R}_2](0)$=200 nM, $[\mathrm{R}_1](0)$=50 nM, and $k_d^{(1)} = k_d^{(2)} = 0.1\ \mathrm{s}^{-1}$.

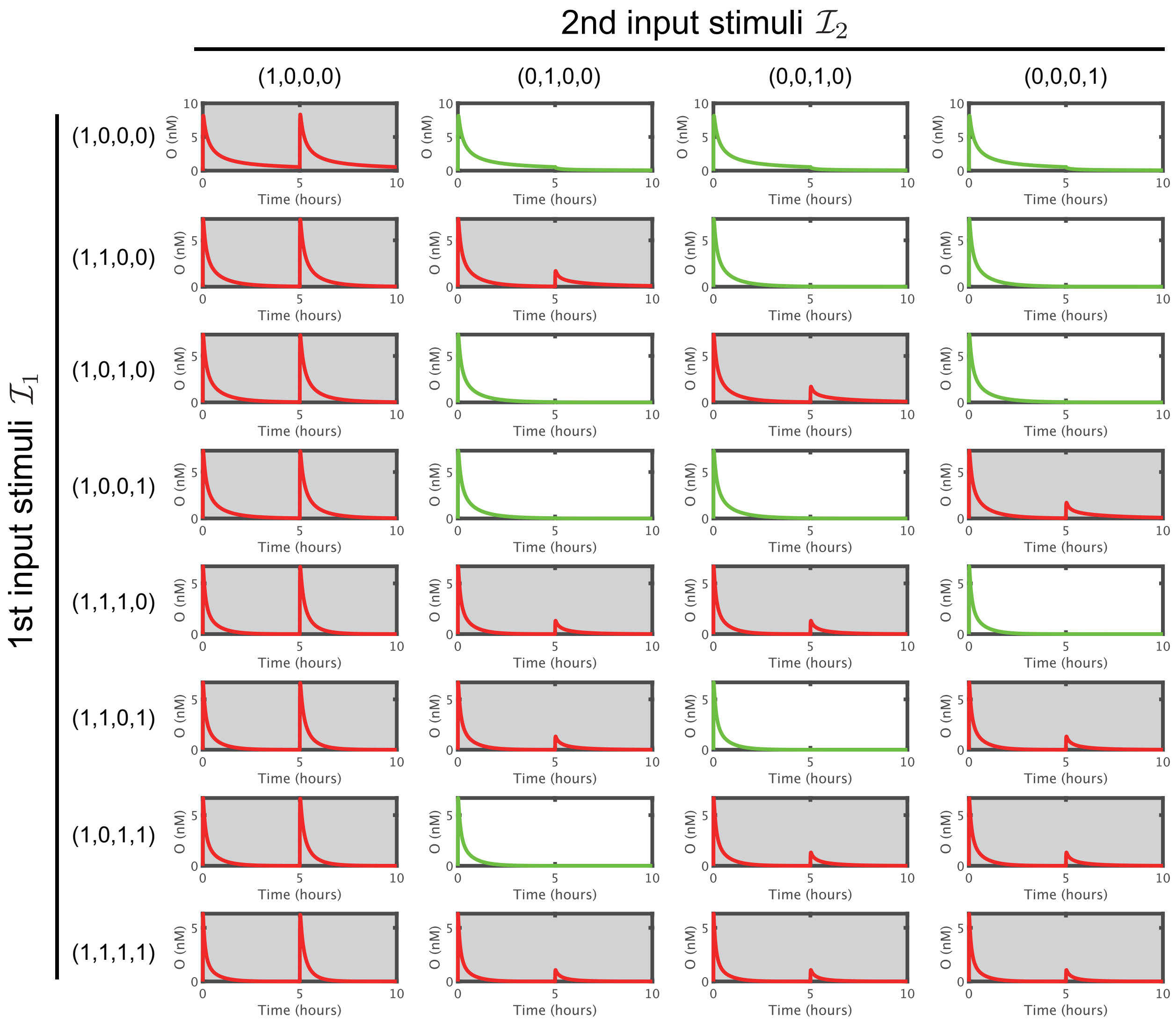

Supporting Figure S11 Simulations of the generalized conditional reflection circuit with 4 input channels. Simulation results of 4-input and 1-output conditioned reflex circuit. Selected 32-input histories are shown, where input histories that were expected to obtain the conditioned reflex are denoted by shadowed boxes, and output responses that were responsive to the second input are denoted by red lines.

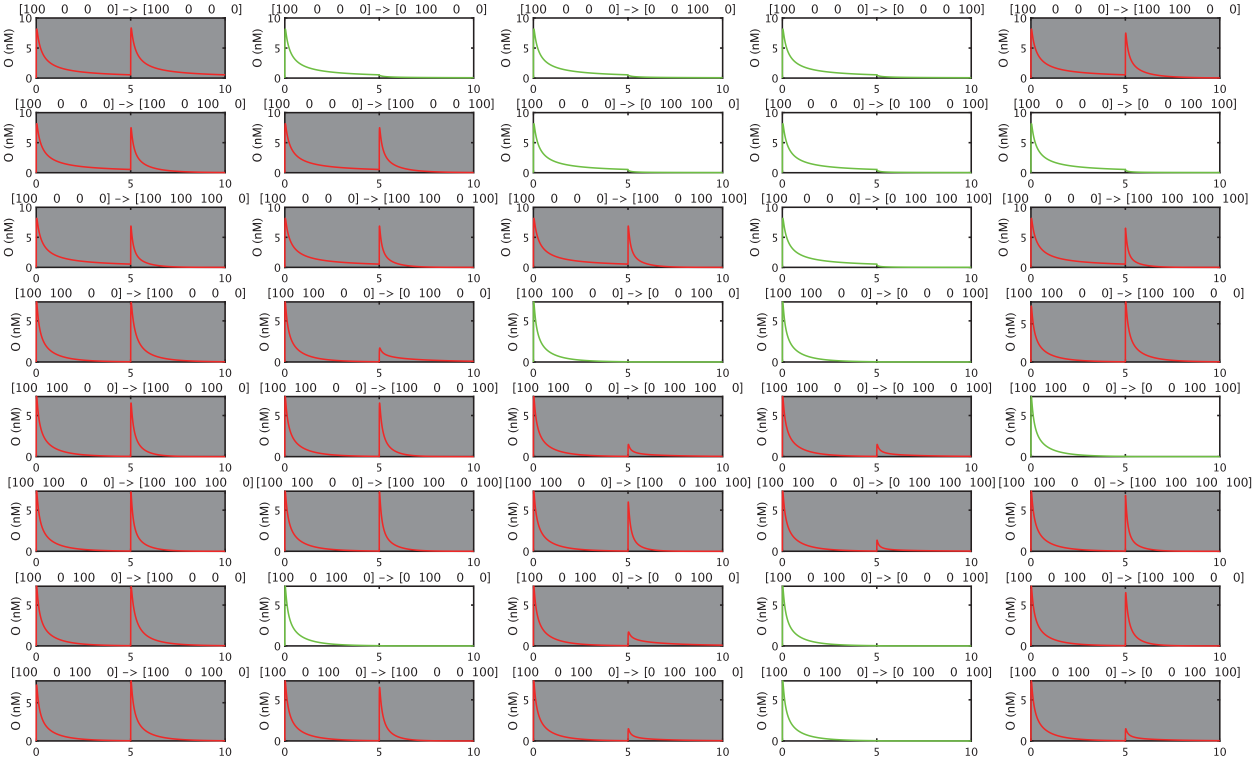

Supporting Figure S12 (Page 1 of 3)

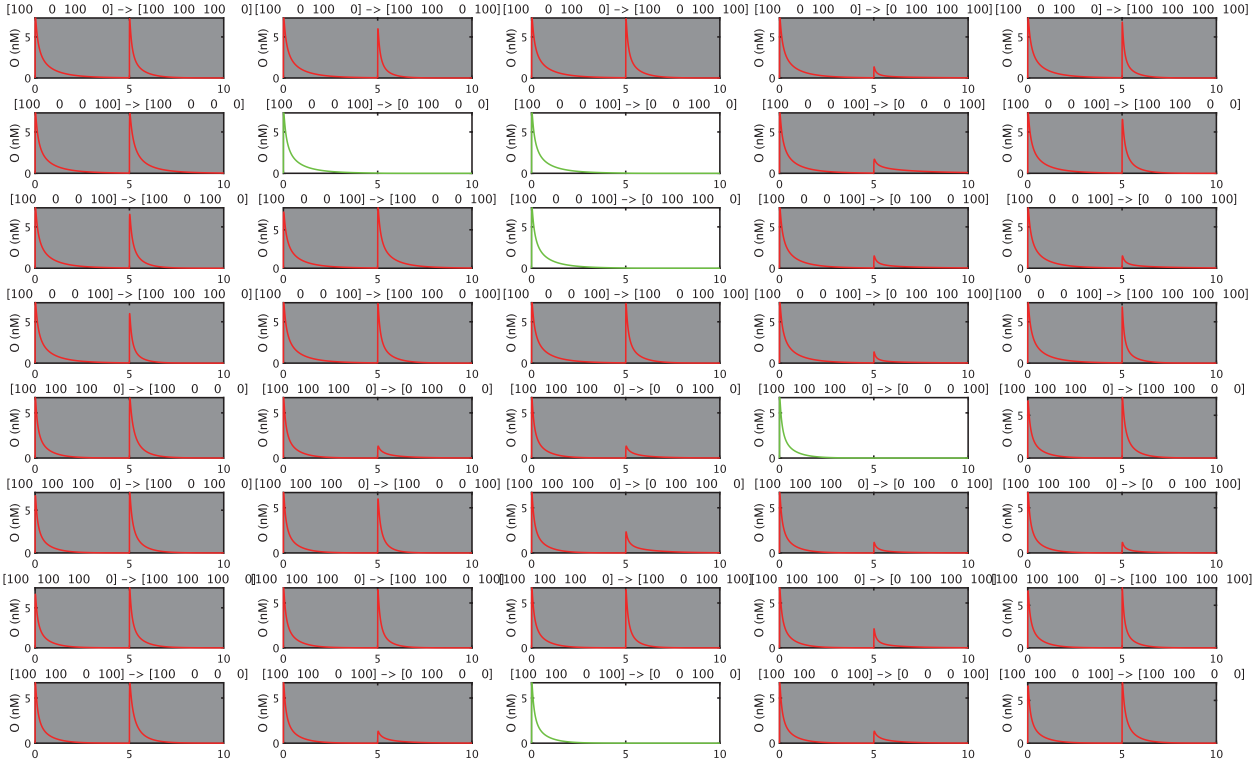

Supporting Figure S12 (Page 2 of 3)

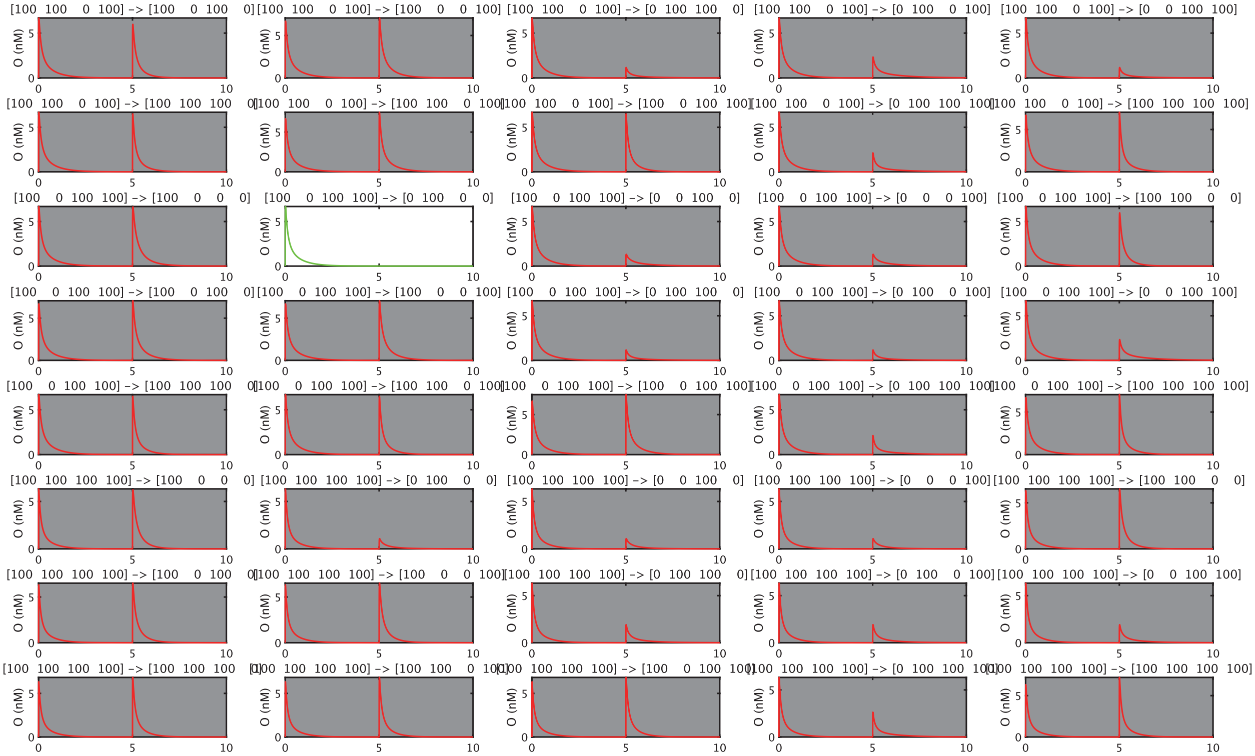

Supporting Figure S12 (Page 3 of 3) Simulation results of 4-input and 1-output conditioned reflex circuit. All 120 input histories are shown, where those expected to obtain the conditioned reflex are denoted by shadowed boxes, and output responses responsive to the second input are denoted by red lines.

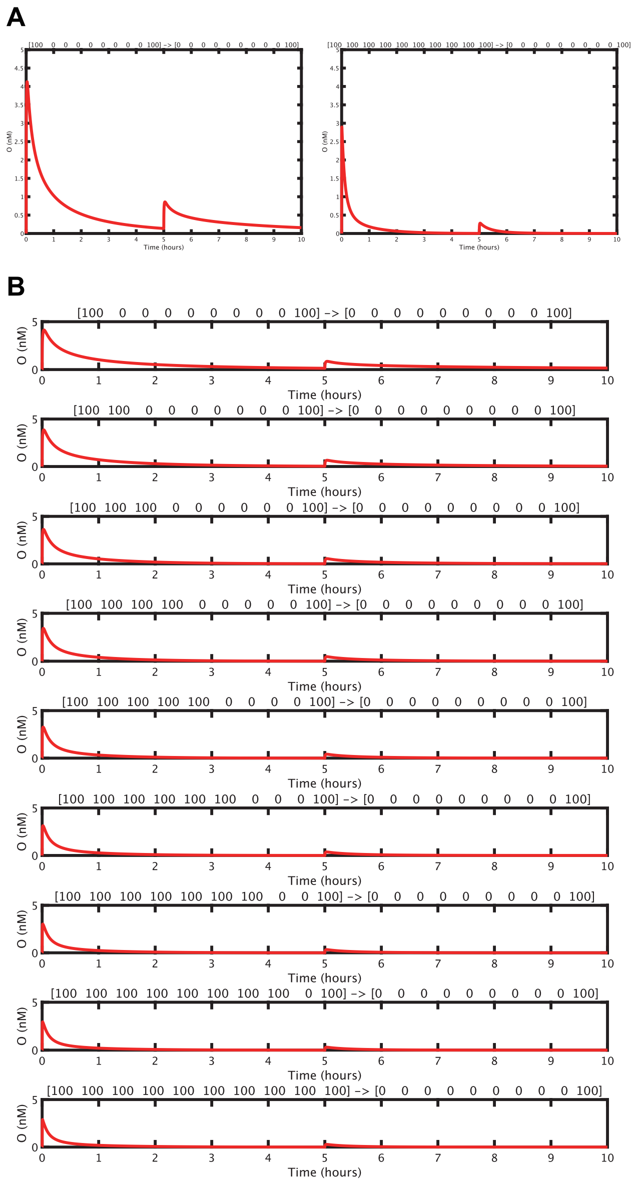

Figure S13 Simulation results of 10-input and 1-output conditioned reflex circuit. The first inputs $\mathcal{I}_1$ such as (1,0,0,0,0,0,0,0,0,1), (1,1,0,0,0,0,0,0,0,1), (1,1,1,0,0,0,0,0,0,1), (1,1,1,1,1,0,0,0,0,1), (1,1,1,1,1,1,0,0,0,1), (1,1,1,1,1,1,1,0,0,1), (1,1,1,1,1,1,1,1,0,1), and (1,1,1,1,1,1,1,1,1,1), and the second input $\mathcal{I}_2 = (0,0,0,0,0,0,0,0,0,1)$ were investigated. (A) Output responses upon $\mathcal{I}_1 = (1,0,0,0,0,0,0,0,0,1)$ and $\mathcal{I}_2 = (0,0,0,0,0,0,0,0,0,1)$ (left) and $\mathcal{I}_1 = (1,1,1,1,1,1,1,1,1,1)$ and $\mathcal{I}_2 = (0,0,0,0,0,0,0,0,0,1)$ (right) are shown. (B) Output responses of all nine input histories are shown.

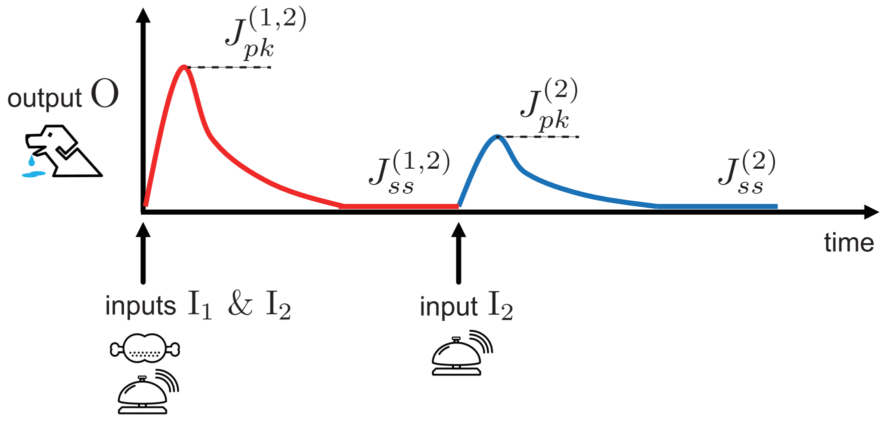

Figure S14 Definition of evaluation values ($J_{pk}^{(1,2)}$, $J_{ss}^{(1,2)}$, $J_{pk}^{(2)}$, and $J_{ss}^{(2)}$) in output responses for designing the cost function of the parameter estimation.

Supporting Text S1 Mathematical modeling of conditioned reflex circuit

Let the state vector $x = [x_1\ x_2\ \cdots\ x_{14}]^T \in R^{14}$ of the conditioned reflex circuit shown in Fig. 2 be defined by:

$$x = [[I_1]\ [M_1]\ [W_1]\ [Y_1]\ [I_2]\ [M_2]\ [W_2]\ [Y_2]\ [M_{2p}]\ [Y_{2p}]\ [S]\ [R_1]\ [R_2]\ [O]]^T, \tag{1}$$

where the concentration of a chemical species "X" in a reaction system is expressed by the brackets [X]. The ordinary differential equation is then based on reaction kinetics as described by:

$$\dot{x} = Vr(t), \tag{2}$$

where the stoichiometric matrix $V \in R^{14\times 12}$ and the reaction rate vector $r : R \to R^{12}$ are given by:

$$V = \begin{bmatrix} -1 & 1 & 0 & 0 & 0 & 0 & 0 & 0 & 0 & 0 & -1 & 0 \\ -1 & 1 & 0 & 0 & 0 & 0 & 0 & 0 & 0 & 0 & 0 & 0 \\ 1 & -1 & 0 & 0 & 0 & 0 & 0 & 0 & 0 & 0 & 0 & 0 \\ 1 & -1 & 0 & 0 & 0 & 0 & -1 & 1 & 0 & 0 & 0 & 0 \\ 0 & 0 & -1 & 1 & -1 & 1 & 0 & 0 & 0 & 0 & 0 & -1 \\ 0 & 0 & -1 & 1 & 0 & 0 & 0 & 0 & 0 & 0 & 0 & 0 \\ 0 & 0 & 1 & -1 & 1 & -1 & 0 & 0 & 0 & 0 & 0 & 0 \\ 0 & 0 & 1 & -1 & 0 & 0 & 0 & 0 & -1 & 1 & 0 & 0 \\ 0 & 0 & 0 & 0 & -1 & 1 & 0 & 0 & 0 & 0 & 0 & 0 \\ 0 & 0 & 0 & 0 & 1 & -1 & 0 & 0 & 0 & 0 & 0 & 0 \\ 0 & 0 & 0 & 0 & 0 & 0 & -1 & 1 & -1 & 1 & 0 & 0 \\ 0 & 0 & 0 & 0 & 0 & 0 & 1 & -1 & 0 & 0 & 0 & 0 \\ 0 & 0 & 0 & 0 & 0 & 0 & 0 & 0 & 1 & -1 & 0 & 0 \\ 0 & 0 & 0 & 0 & 0 & 0 & 1 & -1 & 1 & -1 & 0 & 0 \end{bmatrix}, \tag{3}$$

$$
r = \begin{bmatrix} r_{1_f}^{(1)} \\ r_{1_b}^{(1)} \\ r_{1_f}^{(2)} \\ r_{1_b}^{(2)} \\ \bar{r}_{1_f}^{(2)} \\ \bar{r}_{1_b}^{(2)} \\ r_{2_f}^{(1)} \\ r_{2_b}^{(1)} \\ r_{2_f}^{(2)} \\ r_{2_b}^{(2)} \\ r_d^{(1)} \\ r_d^{(2)} \end{bmatrix} = \begin{bmatrix} k_{1_f}^{(1)} x_1 x_2 \\ k_{1_b}^{(1)} x_3 x_4 \\ k_{1_f}^{(2)} x_5 x_6 \\ k_{1_b}^{(2)} x_7 x_8 \\ \bar{k}_{1_f}^{(2)} x_5 x_9 \\ \bar{k}_{1_b}^{(2)} x_7 x_{10} \\ k_{2_f}^{(1)} x_4 x_{11} \\ k_{2_b}^{(1)} x_{12} x_{14} \\ k_{2_f}^{(2)} x_8 x_{11} \\ k_{2_b}^{(2)} x_{13} x_{14} \\ k_d^{(1)} x_1 \\ k_d^{(2)} x_5 \end{bmatrix}, \qquad (4)
$$

where $k_{i_f}^{(j)}$, $k_{i_b}^{(j)}$, $\bar{k}_{1_f}^{(1)}$, $\bar{k}_{1_b}^{(1)}$, and $k_d^{(j)}$ $(i, j = 1, 2)$ are the reaction rates.

Supporting Text S2 Mathematical modeling of generalized conditioned reflex circuit

Let the state vector $x \in R^{7n+2}$ of the generalized conditioned reflex circuit shown in Fig. 5 be defined by:

$$x = [[S]\ [O]\ [I_1]\ [M_1]\ [W_1]\ [Y_1]\ [M_{p1}]\ [Y_{p1}]\ [R_1]\ \cdots\ [I_n]\ [M_n]\ [W_n]\ [Y_n]\ [M_{pn}]\ [Y_{pn}]\ [R_n]]^T . \tag{5}$$

Then, the ordinary differential equation of the form (2) is given with the stoichiometric matrix $V \in R^{(7n+2)\times 7n}$ and the reaction rate vector $r : R \to R^{7n}$ as:

$$V = \begin{bmatrix} V_0 & V_0 & \cdots & V_0 \\ V_1 & \mathrm{O} & \cdots & \mathrm{O} \\ \mathrm{O} & V_1 & \cdots & \mathrm{O} \\ \vdots & \vdots & \ddots & \vdots \\ \mathrm{O} & \cdots & \cdots & V_1 \end{bmatrix}, \tag{6}$$

$$V_0 = \begin{bmatrix} 0 & 0 & 0 & 0 & 0 & -1 & 1 \\ 0 & 0 & 0 & 0 & 0 & 1 & -1 \end{bmatrix}, \quad V_1 = \begin{bmatrix} -1 & -1 & 1 & -1 & 1 & 0 & 0 \\ 0 & -1 & 1 & 0 & 0 & 0 & 0 \\ 0 & 1 & -1 & 1 & -1 & 0 & 0 \\ 0 & 1 & -1 & 0 & 0 & -1 & 1 \\ 0 & 0 & 0 & -1 & 1 & 0 & 0 \\ 0 & 0 & 0 & 1 & -1 & 0 & 0 \\ 0 & 0 & 0 & 0 & 0 & 1 & -1 \end{bmatrix}, \tag{7}$$

and

$$r = \begin{bmatrix} r_d^{(1)} \\ r_{1_f}^{(1)} \\ r_{1_b}^{(1)} \\ r_{p_f}^{(1)} \\ r_{p_b}^{(1)} \\ r_{2_f}^{(1)} \\ r_{2_b}^{(1)} \\ \vdots \\ r_d^{(n)} \\ r_{1_f}^{(n)} \\ r_{1_b}^{(n)} \\ r_{p_f}^{(n)} \\ r_{p_b}^{(n)} \\ r_{2_f}^{(n)} \\ r_{2_b}^{(n)} \end{bmatrix} = \begin{bmatrix} k_d^{(1)} x_3 \\ k_{1_f}^{(1)} x_3 x_4 \\ k_{1_b}^{(1)} x_5 x_6 \\ k_{p_f}^{(1)} x_3 x_7 \\ k_{p_b}^{(1)} x_5 x_8 \\ k_{2_f}^{(1)} x_1 x_6 \\ k_{2_b}^{(1)} x_2 x_9 \\ \vdots \\ k_d^{(n)} x_{7n-4} \\ k_{1_f}^{(n)} x_{7n-4} x_{7n-3} \\ k_{1_b}^{(n)} x_{7n-2} x_{7n-1} \\ k_{p_f}^{(n)} x_{7n-4} x_{7n} \\ k_{p_b}^{(n)} x_{7n-2} x_{7n+1} \\ k_{2_f}^{(n)} x_1 x_{7n-1} \\ k_{2_b}^{(n)} x_2 x_{7n+2} \end{bmatrix}, \tag{8}$$

where $\mathrm{O} \in R^{7\times 7}$ is the zero matrix, and $k_d^{(i)}$, $k_{*_f}^{(i)}$, and $k_{*_b}^{(i)}$ $(i = 1, 2)$ are the reaction rates.

Based on the optimized parameters obtained for the two-input and one-output conditioned reflex circuit, the initial concentrations were given by $[\mathrm{M}_1](0)$=$[\mathrm{M}_{1i}](0)$=$[\mathrm{R}_i](0)$=[S](0)=100 nM $(i = 1, 2, ..., n)$, and the others were 0 nM; the degradation rates were $k_d^{(i)} = 0.01$ 1/s $(i = 1, 2, ..., n)$, and all strand displacement reaction rates, $k_{i_f}^{(j)}$ and $k_{i_b}^{(j)}$ $(i \in \{1, p, 2\}, j = 1, 2, ..., n)$, were calculated as 7.28×10^{-4} 1/nMs with 164 nM in the critical concentration.

Supporting Text S3 Mathematical modeling of the renewable threshold gate

Let the state vector $x \in R^9$ of the threshold gate in Fig. 8B-C of the main text be defined by

$$x = [[O]\ [T_t]\ [T_p]\ [T_q]\ [T_g]\ [Z]\ [T_i]\ [T_f]\ [T_w]]^T\,. \tag{9}$$

The ordinary differential equation of the form $\dot{x} = Vr$ under BL irradiation condition is then given with the stoichiometric matrix $V \in R^{9\times 6}$ and the reaction rate vector $r : R \to R^6$ as follows:

$$V = \begin{bmatrix} -1 & 1 & -1 & 1 & 1 & -1 \\ -1 & 1 & 0 & 0 & 0 & 0 \\ 1 & -1 & 0 & 0 & 0 & 0 \\ 1 & -1 & 0 & 0 & 0 & 0 \\ 0 & 0 & -1 & 1 & 0 & 0 \\ 0 & 0 & 1 & -1 & 0 & 0 \\ 0 & 0 & 1 & -1 & -1 & 1 \\ 0 & 0 & 0 & 0 & -1 & 1 \\ 0 & 0 & 0 & 0 & 1 & -1 \end{bmatrix}, \tag{10}$$

and

$$r = \begin{bmatrix} r_{1_f}^{(T)} \\ r_{1_b}^{(T)} \\ r_{2_f}^{(T)} \\ r_{2_b}^{(T)} \\ r_{3_f}^{(T)} \\ r_{3_b}^{(T)} \end{bmatrix} = \begin{bmatrix} k_{1_f}^{(T)} x_1 x_2 \\ k_{1_b}^{(T)} x_3 x_4 \\ k_{2_f}^{(T)} x_1 x_5 \\ k_{2_b}^{(T)} x_6 x_7 \\ k_{3_f}^{(T)} x_7 x_8 \\ k_{3_b}^{(T)} x_1 x_9 \end{bmatrix}, \tag{11}$$

where $k_{i_f}^{(T)}$, and $k_{i_b}^{(T)}$ $(i = 1, 2, 3)$ are the reaction rates.

The initial concentrations were given by $[T_t](0)=[T_g](0)=[T_f](0)=100$ nM, and the others were 0 nM. The reaction rates (1/nMs) were calculated according to the calculation method,[2]

and given by $k_{1_f}^{(T)} = 2.7 \times 10^{-3}$ and $k_{1_b}^{(T)} = 0$ based on 6 nt-toehold mediated irreversible reaction, and $k_{2_f}^{(T)} = k_{2_b}^{(T)} = k_{3_f}^{(T)} = k_{3_b}^{(T)} = 7.28 \times 10^{-4}$ based on 5 nt-toehold mediated reversible reactions, where the lengths of the recognition domains were fixed by 20 nt, and temperature was assumed to be 25 °C.

Meanwhile, under UV irradiation conditions, a balance between the forward and backward flows of each reaction is altered due to the obstruction by the cis-type azobenzene. The ordinary differential equation of the form $\dot{x} = V\bar{r}$ under UV irradiation condition is then given with the stoichiometric matrix $V \in R^{9\times 7}$ and the reaction rate vector $r : R \to R^7$ as follows:

$$V = \begin{bmatrix} -1 & 1 & -1 & 1 & 1 & -1 & 0 \\ -1 & 1 & 0 & 0 & 0 & 0 & 0 \\ 1 & -1 & 0 & 0 & 0 & 0 & 0 \\ 1 & -1 & 0 & 0 & 0 & 0 & 0 \\ 0 & 0 & -1 & 1 & 0 & 0 & 1 \\ 0 & 0 & 1 & -1 & 0 & 0 & -1 \\ 0 & 0 & 1 & -1 & -1 & 1 & 0 \\ 0 & 0 & 0 & 0 & -1 & 1 & 1 \\ 0 & 0 & 0 & 0 & 1 & -1 & -1 \end{bmatrix}, \tag{12}$$

and

$$\bar{r} = \begin{bmatrix} \bar{r}_{1_f}^{(T)} \\ \bar{r}_{1_b}^{(T)} \\ \bar{r}_{2_f}^{(T)} \\ \bar{r}_{2_b}^{(T)} \\ \bar{r}_{3_f}^{(T)} \\ \bar{r}_{3_b}^{(T)} \\ \bar{r}_{4_f}^{(T)} \end{bmatrix} = \begin{bmatrix} \bar{k}_{1_f}^{(T)} x_1 x_2 \\ \bar{k}_{1_b}^{(T)} x_3 x_4 \\ \bar{k}_{2_f}^{(T)} x_1 x_5 \\ \bar{k}_{2_b}^{(T)} x_6 x_7 \\ \bar{k}_{3_f}^{(T)} x_7 x_8 \\ \bar{k}_{3_b}^{(T)} x_1 x_9 \\ \bar{k}_{4_f}^{(T)} x_6 x_9 \end{bmatrix}, \tag{13}$$

where new reaction $r_{4_f}^{(T)}$ is added.

The reaction rates (1/nMs) were given by $\bar{k}_{1_f}^{(T)} = \bar{k}_{2_f}^{(T)} = 0$ and $\bar{k}_{1_b}^{(T)} = \bar{k}_{2_b}^{(T)} = \bar{k}_{4_f}^{(T)} = 2.7 \times 10^{-3}$ based on 6 nt-toehold mediated irreversible reaction, and $\bar{k}_{3_f}^{(T)} = \bar{k}_{3_b}^{(T)} = 0$.

Supporting Text S4 Optimization of learning efficiency

The accumulation of memory gate M_2 in the learning condition is an essential condition for learning. In this study, we investigated how the reaction rates in the circuit were related to the updating of the memory gate by evaluating the steady state of the memory gate M_2 upon simultaneous I_1 and I_2 inputs. To this end, we used a structural sensitivity analysis, a straightforward method for performing steady-state analysis for chemical reaction systems.[3]

For the state vector (1) and the ordinary differential equations (2) of the conditioned reflex circuit, the corresponding augmented matrix $A \in R^{19\times 19}$ is derived by:

$$A = \left[\begin{array}{ccc|ccc} \frac{\partial r_1}{\partial x_1} & \cdots & \frac{\partial r_1}{\partial x_{14}} & & & \\ \vdots & \ddots & \vdots & -c^1 & \cdots & -c^5 \\ \frac{\partial r_{12}}{\partial x_1} & \cdots & \frac{\partial r_{12}}{\partial x_{14}} & & & \\ \hline & -(d^1)^T & & 0 & \cdots & 0 \\ & \vdots & & \vdots & & \vdots \\ & -(d^7)^T & & 0 & \cdots & 0 \end{array}\right], \tag{14}$$

where $c^n \in R^{12}$ $(n = 1, ..., 5)$ and $d^n \in R^{14}$ $(n = 1, ..., 7)$ are the basis vectors of the right and left null space of V, respectively, that is, $\mathrm{Ker}(V)$ and $\mathrm{Ker}(V^T)$:

$$
\begin{aligned}
c_1 &= \begin{bmatrix} 0 & 0 & 0 & 0 & 0 & 0 & 0 & 0 & 1 & 1 & 0 & 0 \end{bmatrix}^T, \\
c_2 &= \begin{bmatrix} 0 & 0 & 0 & 0 & 0 & 0 & 1 & 1 & 0 & 0 & 0 & 0 \end{bmatrix}^T, \\
c_3 &= \begin{bmatrix} 0 & 0 & 0 & 0 & 1 & 1 & 0 & 0 & 0 & 0 & 0 & 0 \end{bmatrix}^T, \\
c_4 &= \begin{bmatrix} 0 & 0 & 1 & 1 & 0 & 0 & 0 & 0 & 0 & 0 & 0 & 0 \end{bmatrix}^T, \\
c_5 &= \begin{bmatrix} 1 & 1 & 0 & 0 & 0 & 0 & 0 & 0 & 0 & 0 & 0 & 0 \end{bmatrix}^T, \\
d_1 &= \begin{bmatrix} 0 & 1 & 0 & 1 & 0 & 1 & 0 & 1 & 0 & 0 & 0 & 0 & 0 & 1 \end{bmatrix}^T, \\
d_2 &= \begin{bmatrix} 0 & 0 & 0 & 0 & 0 & 1 & 0 & 1 & 0 & 0 & 0 & 0 & 1 & 0 \end{bmatrix}^T, \\
d_3 &= \begin{bmatrix} 0 & 1 & 0 & 1 & 0 & 0 & 0 & 0 & 0 & 0 & 0 & 1 & 0 & 0 \end{bmatrix}^T, \\
d_4 &= \begin{bmatrix} 0 & 1 & 0 & 1 & 0 & 1 & 0 & 1 & 0 & 0 & 1 & 0 & 0 & 0 \end{bmatrix}^T, \\
d_5 &= \begin{bmatrix} 0 & 0 & 0 & 0 & 0 & 1 & 1 & 0 & 0 & 1 & 0 & 0 & 0 & 0 \end{bmatrix}^T, \\
d_6 &= \begin{bmatrix} 0 & 0 & 0 & 0 & 0 & 1 & 1 & 0 & 1 & 0 & 0 & 0 & 0 & 0 \end{bmatrix}^T, \\
d_7 &= \begin{bmatrix} 0 & 1 & 1 & 0 & 0 & 0 & 0 & 0 & 0 & 0 & 0 & 0 & 0 & 0 \end{bmatrix}^T.
\end{aligned} \tag{15}
$$

Then, the (i, j) element of $-A^{-1}$ reportedly provides the information on how the perturbation of the reaction r_j influences the steady-state concentration of x_i.[3] In the conditioned reflex circuit, we are interested in how effective the steady-state concentration of x_6, that is $[\mathrm{M}_2](\infty)$, can be updated, which can be estimated by assessing the $(6, *)$ element of $-A^{-1}$. Subsequently, we analytically obtained the sixth row vector of $-A^{-1}$ as:

$$
\begin{bmatrix} + & - & - & + & + & - & + & - & - & + & - & - \end{bmatrix}, \tag{16}
$$

where the notations "+" and "-" indicate that the steady-state of x_6 increases or decreases, respectively, when the reaction rate r_j $(j = 1, ..., 12)$ is increased. The analytic calculation was performed by Mathematica (Wolfram Research), and the code is available on GitHub at https://github.com/SYSBIOKYUTECH/Mathematica-code-for-SSA. Based on the reaction rate vector (13), we can see that increasing the reactions $r_{1_f}^{(1)}$, $r_{1_b}^{(2)}$, $\bar{r}_{1_f}^{(2)}$, $r_{2_f}^{(1)}$, and $r_{2_b}^{(2)}$ contributes to an increase in the memory gate M_2, whereas the others have negative effects. To increase reaction $r_{1_f}^{(1)}$, we can either increase the reaction rate $k_{1_f}^{(1)}$ or the initial concentration of the memory gate M_1. In contrast, to increase reaction $r_{1_b}^{(2)}$, increasing the reaction rate $k_{1_b}^{(2)}$ simultaneously induces a negative effect of reaction $\bar{r}_{1_b}^{(2)}$, because these reactions share the toehold domain, t_a. Alternatively, increasing the initial concentration of $x_7(0)$ ($[\mathrm{W}_2](0)$) and/or $x_8(0)$ ($[\mathrm{Y}_2](0)$) is not acceptable as they can induce an unwanted accumulation of M_2 in the non-learning condition. Consequently, increasing reaction $r_{1_b}^{(2)}$ is not adequate. Based on such a consideration, we can conclude that increasing the reaction rate constant $k_{1_f}^{(1)}$ and the initial concentrations of gates M_1, M_{2p}, and R_2 contributes to efficiently updating the memory gate M_2 in the learning condition. The update width of the memory gate in the parameter set P_2 in the learning condition is approximately twice as large as that in the parameter set P_1, which is consistent with the results of the structural sensitivity analysis (Fig. S15). However, the output peak in the post-learning condition remained relatively unchanged. This supports the validity of the optimization in which the initial concentrations and reaction rate parameters were optimized to maximize learning efficiency based on the evaluation function equation $J(x(0), p)$ of the main text.

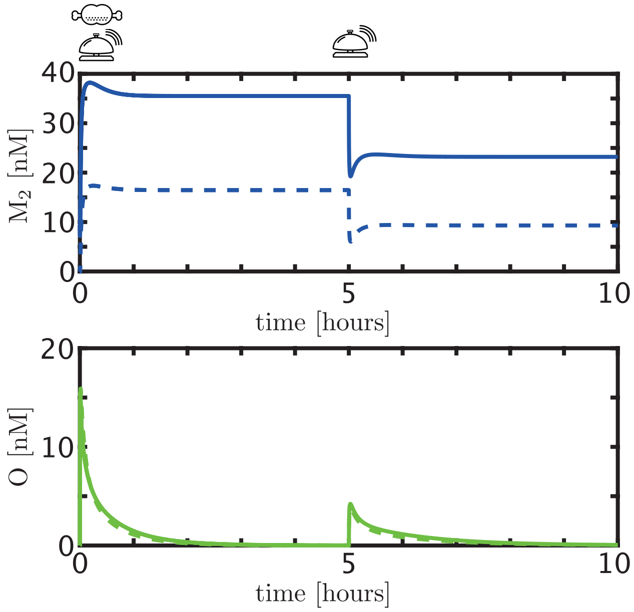

Figure S15 Efficiency in learning conditions. The simulation results with the parameter set P_1 ($[M_1](0)=[M_{2p}](0)=[R_2](0)=100$ nM, $[S](0)=100$ nM, and $[R_1](0)=50$ nM) and P_2 ($[M_1](0)=[M_{2p}](0)=[R_2](0)=200$ nM, $[S](0)=100$ nM, and $[R_1](0)=50$ nM) are denoted by dashed and solid lines, respectively.

References

(1) Lakin, M. R.; Youssef, S.; Polo, F.; Emmott, S.; Phillips, A. Visual DSD: a design and analysis tool for DNA strand displacement systems. *Bioinformatics* **2011**, *27*, 3211–3.

(2) Zhang, D. Y.; Winfree, E. Control of DNA strand displacement kinetics using toehold exchange. *J Am Chem Soc* **2009**, *131*, 17303–14.

(3) Mochizuki, A. A structural approach to understanding enzymatic regulation of chemical reaction networks. *Biochem J* **2022**, *479*, 1265–1283.
